# EpCAM deficiency causes the premature ageing of intestinal stem cells via EGFR/SP1/mTORC1 pathway

**DOI:** 10.1002/ctm2.70219

**Published:** 2025-02-12

**Authors:** Keying Li, Changlong Xu, Lulu Liu, Yunjuan Wang, Jun Chen, Chunyuan Li, Zitong Peng, Xiaoqian Li, Gang Chang, Zili Lei, Yanhong Yang

**Affiliations:** ^1^ Guangdong Metabolic Diseases Research Center of Integrated Chinese and Western Medicine Guangdong Pharmaceutical University Guangzhou P.R. China; ^2^ The Reproductive Medical Center of Nanning Second People's Hospital Nanning P.R. China; ^3^ The First Affiliated Hospital (School of Clinical Medicine) Guangdong Pharmaceutical University Guangzhou P.R. China; ^4^ Department of Biochemistry and Molecular Biology Shenzhen University Medical School Shenzhen P.R. China

1

Dear Editor,

We reported a precise molecular mechanism underlying the longevity of intestinal stem cells (ISCs) maintained by EpCAM via the EGFR/SP1/mTORC1 pathway. EpCAM is highly expressed in ISCs,^1^ and most patients of congenital tufting enteropathy (CTE), caused by EpCAM mutations,^2^, ^3^ die of intestinal failure.^4^ EpCAM deficiency induces premature ageing of ISCs via downregulating TSC1 to hyperactivate the mTORC1 pathway.^5^ However, upstream signals through which EpCAM regulates the expression of TSC1 remain unknown.

As previous report,^5^ EpCAM^−/−^ mice showed no significant differences in appearance compared to wild type (WT) littermates at the E18.5 stage (Figure S1A,B). However, the mutant pups started to have diarrhoea after birth and most died within 1 week. The tufting and erosion of the intestinal villi were observed in mutants at both E18.5 and P3 stages (Figures S1C,D and S2A,B). *Tert*, encoding the protein component of telomerase, was significantly downregulated but γH2AX, the DNA damage accumulation‐associated protein, was considerably increased in the E18.5 mutant small intestines (Figure S1E−G), confirming the premature ageing of these tissues.

SP1 is involved in various tissue and cellular senescence processes.^6^ At the E18.5 stage, the expression and activity of SP1 were reduced in the mutant small intestines (Figure 1A,B). The p‐SP1 was predominantly present in the nucleus of epithelial cells in the inter villi region of the duodenum and decreased in the mutants at both E18.5 and P3 stages (Figures S3A and S4A−F).

**FIGURE 1:**
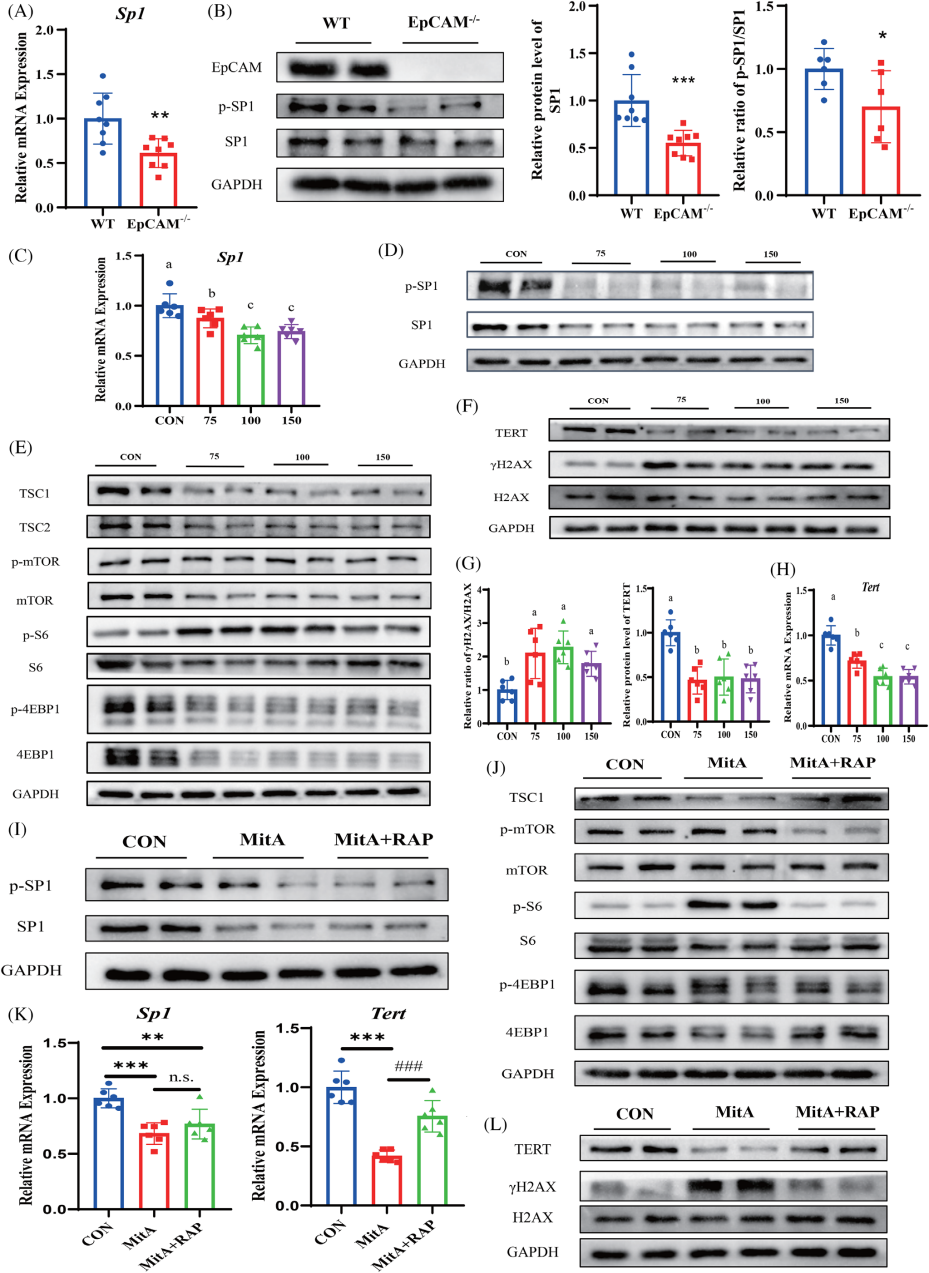
The decrease of the activity of the transcriptional factor SP1 caused the ageing of intestinal epithelial cells both in vivo and in vitro. (A) The relative mRNA expression levels of *Sp1* in the small intestines of WT and EpCAM^−/−^ mice at E18.5 stage (*n* = 8, ^**^ *p* < .01 compared with WT group). (B) Western blot results showed protein levels of EpCAM, p‐SP1 and SP1 in the small intestines of WT and EpCAM^−/−^ mice at E18.5 stage; Right Panel: quantification data (*n* = 6, ^*^ *p* < .05 and ^***^ *p* < .001 compared with WT group). (C) The relative mRNA expression levels of *Sp1* in Caco‐2 cells from CON, 75, 100 and 150 groups (*n* = 6, a, b, c indicate significant differences between groups labelling different letters, and the order of the letters a, b, c also refers to the relative quantity from high to low). (D) Representative western blot images showing protein levels of p‐SP1 and SP1 in Caco‐2 cells from CON, 75, 100 and 150 groups. (E) Representative western blot images showing protein levels of the components of mTORC1 pathway including TSC1, TSC2, p‐mTOR, mTOR, p‐S6, S6, p‐4EBP1 and 4EBP1 in Caco‐2 cells from CON, 75, 100 and 150 groups. (F) Representative western blot images showing protein levels of TERT, γH2AX and H2AX in Caco‐2 cells from CON, 75, 100 and 150 groups. (G) The quantification data results of western blots in Figure F (*n* = 6, a, b indicate significant differences between different groups labelling different letters, and the order of the letters a, b also refers to the relative quantity from high to low). (H) The relative mRNA expression levels of *Tert* in Caco‐2 cells from CON, 75, 100 and 150 groups (*n* = 6, a, b, c indicate significant differences between groups labelling different letters, and the order of the letters a, b, c also refers to the relative quantity from high to low). (I) Representative western blot images showing protein levels of p‐SP1 and SP1 in Caco‐2 cells from each group of CON, MitA and MitA + RAP. (J) Representative western blot images showing protein levels of the components of the mTORC1 pathway including TSC1, p‐mTOR, mTOR, p‐S6, S6, p‐4EBP1 and 4EBP1 in Caco‐2 cells from each group of CON, MitA and MitA + RAP. (K) The relative mRNA expression levels of *Sp1* and *Tert* in Caco‐2 cells from CON, MitA and MitA + RAP groups, respectively (*n* = 6, ^**^ *p* < .01 and ^***^ *p* < 0.001 compared with CON group, ^####^ *p* < 0.001 compared with MitA group, n.s. indicates non‐significant statistical differences). (L) Representative western blot images showing protein levels of TERT, γH2AX and H2AX in Caco‐2 cells from each group of CON, MitA and MitA + RAP. CON, control; 75, 100 and 150 indicate MitA concentrations of 75, 100 and 150  nM, respectively, in Caco‐2 cell cultures; MitA, Mithramycin A; RAP, rapamycin.

The SP1 inhibitor Mithramycin A (MitA) reduced the expression and activity of SP1 in Caco‐2 cells (Figures 1C,D and S3B,C). With the inhibition of SP1, TSC1 and TSC2 downregulated, and the activation of the mTORC1 pathway dramatically increased (Figures 1E and S3D). The TERT significantly decreased, but γH2AX increased with the MitA administration (Figure 1F−H).

Subsequently, Caco‐2 cells were simultaneously treated with MitA and rapamycin (RAP). The p‐SP1 and SP1 were significantly decreased in MitA and MitA+RAP groups (Figures 1I,K and S3E). In the MitA+RAP group, the activation of the mTORC1 pathway was markedly lower than in the MitA group (Figures 1J and S3E). The TERT was increased but γH2AX was decreased in the MitA+RAP group compared with the MitA group (Figures 1K,L and S3E).

The naringenin (NAR), an effective activator of SP1,^7^ was administrated to the pregnant females to activate SP1 in the intestines of EpCAM^−/−^ embryos (Figure S5A). The duodenum and jejunum showed improved intestinal villi tufting and breakage in the NAR‐treated mutants (Figure S5B). The expression and activity of SP1 were significantly restored in the small intestines of the EpCAM^−/−^+NAR group (Figures 2A,B and S5C). After administration of NAR, TSC1 was significantly upregulated but the activation of the mTORC1 pathway noticeably decreased in the mutant intestines (Figures 2C,D and S5D). With NAR treatment, there was a certain tendency for a rebound of the transcription of *Tert* in the mutant intestines (*p* = 0 .07) (Figure 2E). Again, TERT was significantly increased but γH2AX was decreased in the small intestines of NAR‐treated EpCAM^−/−^ mice compared with control mutants (Figures 2F and S5C).

**FIGURE 2:**
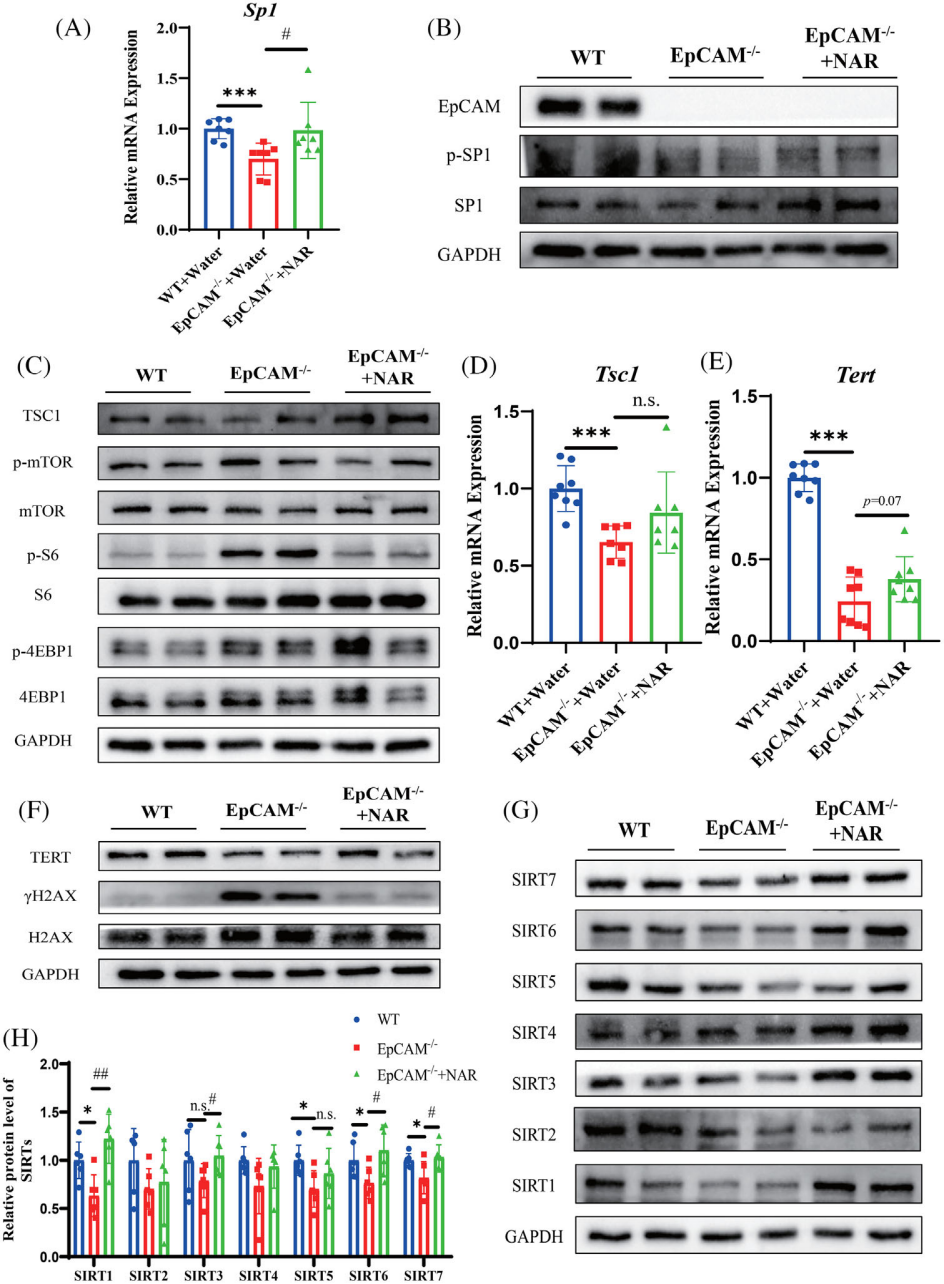
The administration of naringenin improved the premature ageing of intestinal epithelium in EpCAM‐deficient mice via activating the transcriptional factor SP1. (A) The relative mRNA expressional levels of *Sp1* in the small intestines of mice from the WT, EpCAM^−/−^ and EpCAM^−/−^ +NAR groups at E18.5 stage (*n* = 7, ^***^ *p* < 0.001 compared with WT group and ^#^ *p* < .05 compared with EpCAM^−/−^ group). (B) Representative western blot images showing protein levels of EpCAM, p‐SP1 and SP1 in the small intestines of mice from the WT, EpCAM^−/−^ and EpCAM^−/−^ +NAR groups at E18.5 stage. (C) Representative western blot images showing protein levels of the components of mTORC1 pathway including TSC1, p‐mTOR, mTOR, p‐S6, S6, p‐4EBP1 and 4EBP1 in the small intestines of mice from the WT, EpCAM^−/−^ and EpCAM^−/−^ +NAR groups at E18.5 stage. (D) The relative mRNA expression levels of *Tsc1* in the small intestines of mice from the WT, EpCAM^−/−^ and EpCAM^−/−^ +NAR groups at E18.5 stage (*n* = 7, ^***^ *p* < .001 compared with WT group, n.s. indicates non‐significant statistical differences). (E) The relative mRNA expression levels of *Tert* in the small intestines of mice from the WT, EpCAM^−/−^ and EpCAM^−/−^ +NAR groups at E18.5 stage (*n* = 8, ^***^ *p* < .001 compared with WT group). (F) Representative western blot images showing protein levels of TERT, γH2AX and H2AX in the small intestines of mice from the WT, EpCAM^−/−^ and EpCAM^−/−^ +NAR groups at E18.5 stage. (G) Representative western blot images showing protein levels of SIRT1, SIRT2, SIRT3, SIRT4, SIRT5, SIRT6 and SIRT7 in the small intestines of mice from the WT, EpCAM^−/−^ and EpCAM^−/−^ +NAR groups at E18.5 stage. (H) The quantification data of western blot in Figure G (*n* = 6, ^*^ *p* < 0.05 compared with WT group, ^#^ *p* < .05 and ^##^ *p* < .01 compared with EpCAM^−/−^ group, respectively; n.s. indicates non‐significant statistical differences). NAR, naringenin.

Sirtuins decrease in various ageing cells and tissues.^8^ The NAR significantly rescued SIRT1, SIRT3, SIRT6 and SIRT7 in the mutant intestines (Figure 2G,H). The transcriptions of *Sirt3*, *Sirt4*, *Sirt5* and *Sirt7* were significantly restored in the mutant small intestines with the NAR administration (Figure S5E).

In tumour cells of epithelial origin, interactions between EGFR and EpCAM have been demonstrated.^9^ The expression and activity of EGFR were increased in the mutant small intestines at the E18.5 stage (Figure 3A,B). Therefore, gefitinib was selected to reduce the hyperactivation of EGFR in the intestines of EpCAM^−/−^ mice. Gefitinib significantly decreased the ratio of p‐EGFR/EGFR in the small intestines of EpCAM^−/−^ mice (Figures 3C,D and S6A). The TERT was increased in the gefitinib‐administered EpCAM^−/−^ mice, and the γH2AX was reduced accordingly (Figure 3E−G).

**FIGURE 3:**
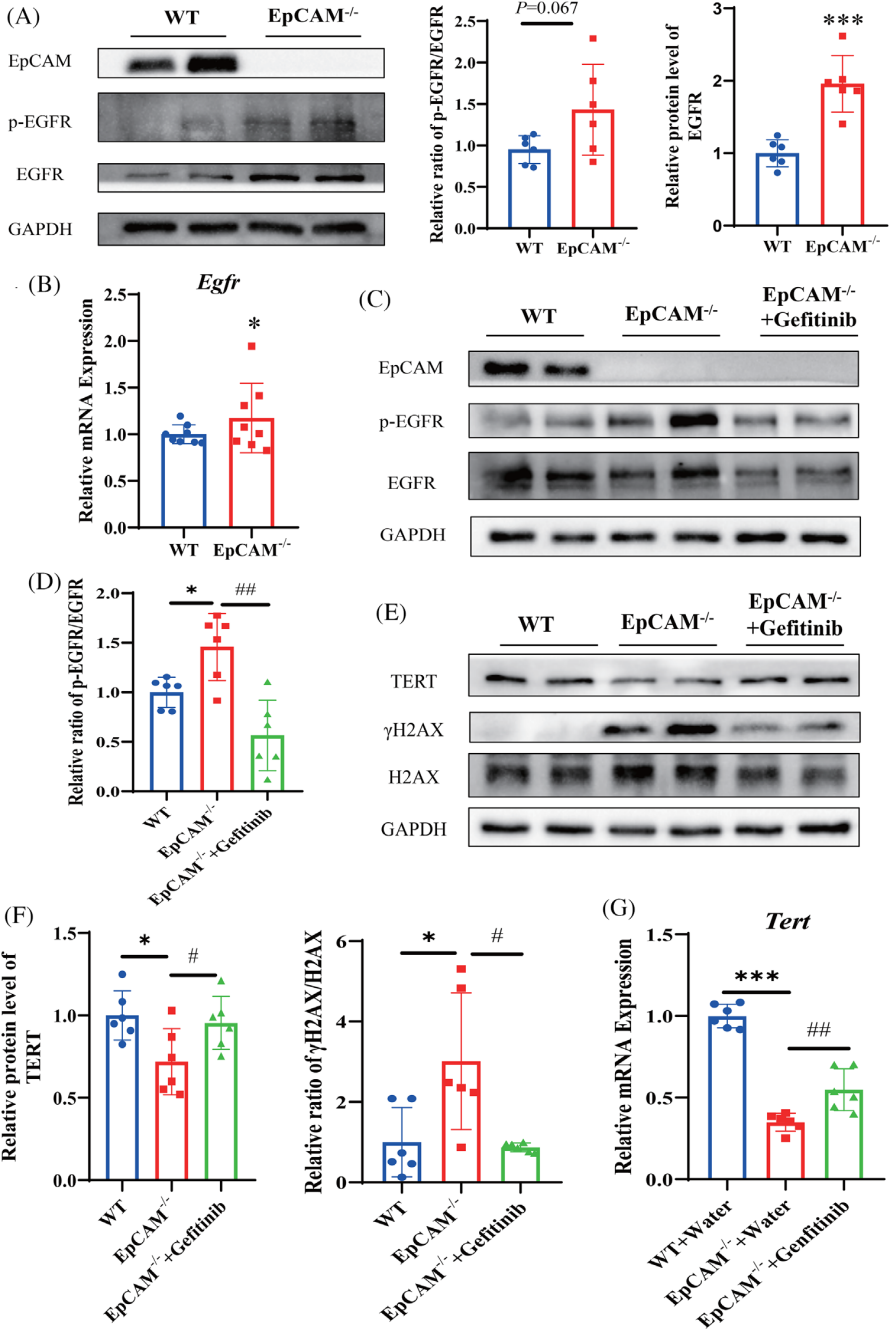
The hyperactivation of EGFR caused the premature ageing of the intestinal epithelium in the EpCAM‐deficient mice. (A) Western blot results showed protein levels of EpCAM, p‐EGFR and EGFR in the small intestines of WT and EpCAM^−/−^ mice at E18.5 stage; Right Panel: quantification data (*n* = 6, ^***^ *p* < .001 compared with WT group). (B) The relative mRNA expression levels of *Egfr* in the small intestines of WT and EpCAM^−/−^ mice at E18.5 stage (*n* = 12, ^*^ *p* < .05 compared with WT group). (C) Representative western blot images showing protein levels of EpCAM, p‐EGFR and EGFR in the small intestines of mice from WT, EpCAM^−/−^ and EpCAM^−/−^ + Gefitinib groups at E18.5 stage. (D) The quantification data of western blot results in Figure C (*n* = 6, ^*^ *p* < .05 compared with WT group, ^###^ *p* < .001 compared with EpCAM^−/−^ group). (E) Representative western blot images showing protein levels of TERT, γH2AX and H2AX in the small intestines of mice from WT+ Water, EpCAM^−/−^ + Water and EpCAM^−/−^ + Gefitinib groups at E18.5 stage. (F) The quantification data of western blot results in Figure E (*n* = 6, ^*^ *p* < .05 compared with WT group, ^#^ *p* < .05 compared with EpCAM^−/−^ group). (G) The relative mRNA expression levels of *Tert* in the small intestines of mice from WT, EpCAM^−/−^ and EpCAM^−/−^ + Gefitinib groups at E18.5 stage (*n* = 6, ^***^ *p* < .001 compared with WT group, ^###^ *p* < .001 compared with EpCAM^−/−^ group).

EGFR can activate the mTORC1 pathway.^10^ The expression and activity of SP1 were significantly rebounded in the small intestines of gefitinib‐treated EpCAM^−/−^ mice (Figures 4A,B and S6B). The expression of TSC1 was significantly restored after the administration of gefitinib, but the activation of the mTORC1 pathway decreased in the small intestines of treated mutants (Figures 4C,D and S6C).

**FIGURE 4:**
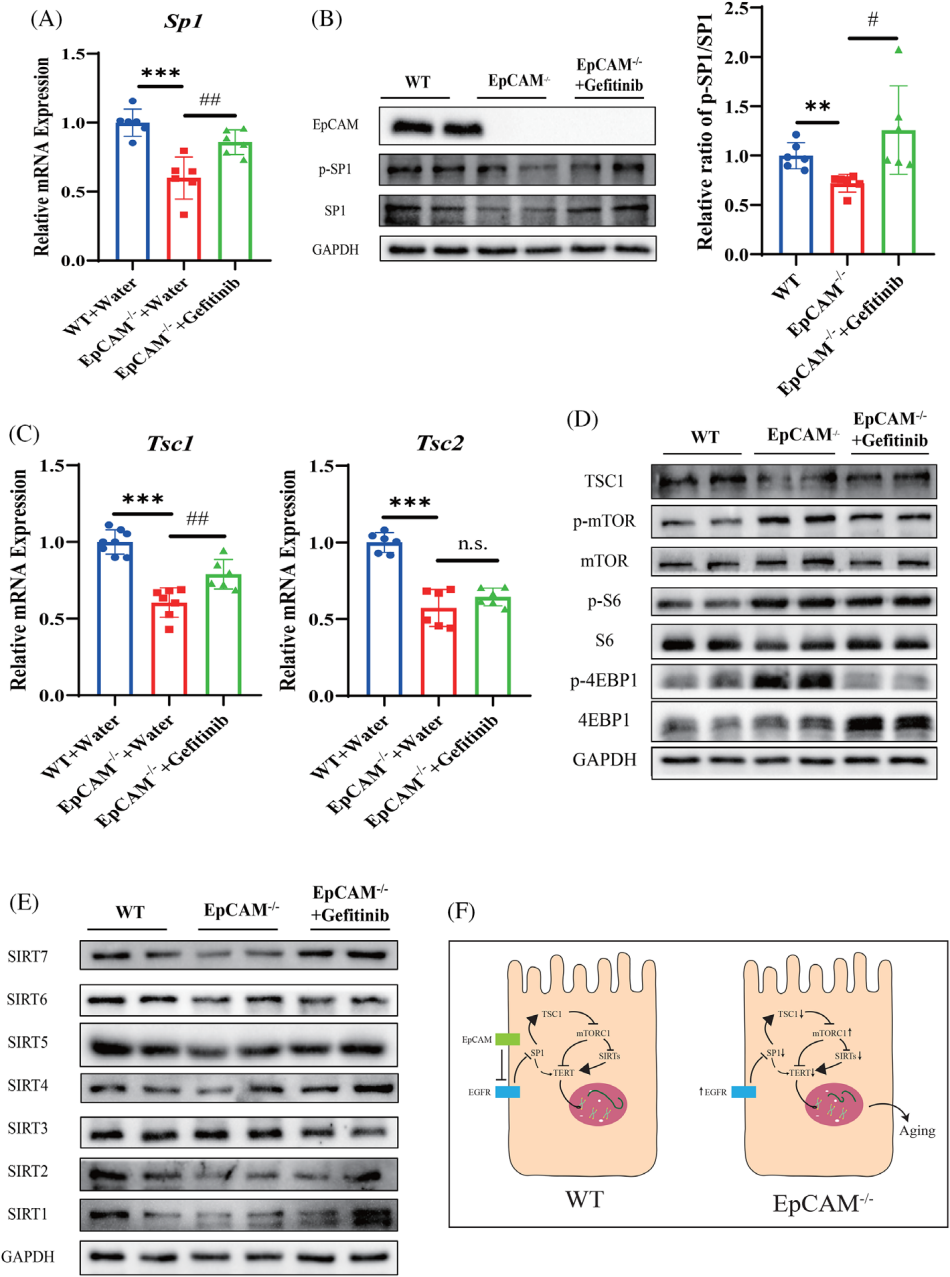
The moderate inhibition of the EGFR activity could effectively improve the hyperactivation of mTORC1 pathway and rescue the downregulations of Sirt family members in the intestinal epithelium of EpCAM‐deficient mice via activating the transcriptional factor SP1. (A) The relative mRNA expression levels of *Sp1* in the small intestines of mice from WT, EpCAM^−/−^ and EpCAM^−/−^ + Gefitinib groups at E18.5 stage (*n* = 6, ^***^ *p* < .001 compared with WT group, ^###^ *p* < .001 compared with EpCAM^−/−^ group). (B) Representative western blot images showing protein levels of EpCAM, p‐SP1 and SP1 in the small intestines of mice from WT, EpCAM^−/−^ and EpCAM^−/−^ + Gefitinib groups at E18.5 stage, and the quantification data for the ratio of p‐SP1/SP1 (*n* = 6, ^**^ *p* < .01 compared with WT group, ^#^ *p* < .05 compared with EpCAM^−/−^ group). (C) The relative mRNA expression levels of *Tsc1* and *Tsc2* in the small intestines of mice from WT, EpCAM^−/−^ and EpCAM^−/−^ + Gefitinib groups at E18.5 stage (*n* = 6, ^***^ *p* < 0.001 compared with WT group, ^###^ *p* < .001 compared with EpCAM^−/−^ group, n.s. indicates non‐significant statistical differences). (D) Representative western blot images showing protein levels of the components of mTORC1 pathway including TSC1, p‐mTOR, mTOR, p‐S6, S6, p‐4EBP1 and 4EBP1 in the small intestines of mice from WT, EpCAM^−/−^ and EpCAM^−/−^ + Gefitinib groups at E18.5 stage. (E) Representative western blot images showing protein levels of SIRT1, SIRT2, SIRT3, SIRT4, SIRT5, SIRT6 and SIRT7 in the small intestines of mice from WT, EpCAM^−/−^ and EpCAM^−/−^ + Gefitinib groups at E18.5 stage. (F) EpCAM deficiency leads to the hyperactivation of EGFR pathway in the intestinal epithelial cells, and then the sustained hyperactivated EGFR pathway causes the reduction of the expression and activity of the transcriptional factor SP1. Following the reduction of the activity of SP1, the expression of *Tsc1* is downregulated, and then the reduced TSC1 causes the hyperactivation of the mTORC1 pathway. Finally, the hyperactivated mTORC1 pathway leads to the premature ageing of intestinal stem cells, showing the ageing phenotypes such as the downregulation of *Tert* expression and decreased genomic stability. Moreover, the reduction of the activity of SP1 might also directly downregulate the expression of *Tert* in the intestinal stem cells.

A significant rebound of SIRT1 and SIRT5 and an increasing trend of SIRT6 and SIRT7 were confirmed in the small intestines of EpCAM^−/−^ mice after administration of gefitinib (Figures 4E and S6D). The transcriptions of *Sirt1*, *Sirt3*, *Sirt4*, *Sirt5* and *Sirt7* were restored in the small intestines of gefitinib‐administrated EpCAM^−/−^ mice (Figure S6E).

In conclusion, loss of EpCAM causes the premature ageing of ISCs via EGFR/SP1/mTORC1 pathway (Figure 4F). EpCAM deficiency causes the hyperactivation of EGFR in the ISCs. Then, the sustained hyperactivation of EGFR decreases the activity of SP1 which controls the expression of *Tsc1*. Subsequently, the downregulated TSC1 induces the hyperactivation of mTORC1 and finally causes the premature ageing of the ISCs. Our findings put forward a new viewpoint on the interactions between EpCAM and EGFR in the membranes of stem cells and even tumour cells and provide potential targets for the CTE therapy.

## AUTHOR CONTRIBUTIONS

K.L.: Methodology, investigation, writing—original draft preparation. C.X.: Investigation, funding acquisition. L.L.: Methodology, validation. Y.W.: Methodology, visualization. J.C.: Software. C.L.: Visualization. Z.P.: Methodology. X.L.: Methodology. G.C.: Writing—review and editing, funding acquisition. Z.L.: Supervision, conceptualization, funding acquisition. Y.Y.: Supervision, investigation, funding acquisition.

## CONFLICT OF INTEREST STATEMENT

The authors report no conflicts of interest.

## FUNDING INFORMATION

This work was supported by the National Natural Science Foundation of China (82171855); the Key Field Special Project for Colleges and Universities of Guangdong Province (Biomedical and Health) (2023ZDZX2030); the Natural Science Foundation of Shenzhen (JCYJ20210324120212033); the Guangdong Basic and Applied Basic Research Foundation (2021A1515012383); the Scientific Research and Technological Development Plan of Nanning City (20213024); the Science and Technology Project of Qing‐Xiu District of Nanning City (2020025); the Science and Technology Project of Jiang‐Nan District of Nanning City (20220620‐8, 20230715‐07); and the Key Research and Development Plan of Scientific Research and Technology in Liang‐Qing District of Nanning City (202213, 202216, 202311).

## ETHICS APPROVAL

All animal experimental procedures were approved by the Experimental Animal Ethics Committee of Guangdong Pharmaceutical University.

## Supporting information



Supporting Information

## Data Availability

Datasets are available from corresponding authors on reasonable request.
