# Supplementary material for: EpCAM deficiency causes the premature ageing of intestinal stem cells via EGFR/SP1/mTORC1 pathway
Source: Clin Transl Med. 2025 Feb 12;15(2):e70219. doi: 10.1002/ctm2.70219 (PMC11821454; doi:10.1002/ctm2.70219)
Supplement: Supplementary file 1 — Supporting Information [file CTM2-15-e70219-s001.docx]

**Supplementary information**

**Index**

**• Materials and methods**

**• Supplemental Figures**

**• Supplemental tables description**

**MATERIALS AND METHODS**

**Mice**

All animal experiments were approved by the Experimental Animal Ethic Committee of Guangdong Pharmaceutical University (Guangzhou, China) and adhered to the ARRIVE1 guidelines ^1^. EpCAM knockout mice were successfully generated by CRISPR/Cas9 technology previously ^2^. All the mice were maintained in C57BL/6 background. Mice were housed in the SPF mouse facility at 25℃, 60-65% humidity and 12hr light-dark cycle, with free access to water and food. Heterozygous males and females were mated to get embryos and pups for experiments. E18.5 embryos were collected from pregnant heterozygous females. Intestinal tissues were harvested from E18.5 embryos and P3 pups respectively. The intestines from EpCAM^-/-^ embryos and pups were studied, and the intestinal tissues from WT embryo and pups were used as control.

Gefitinib (7.5mg/kg; Meilun Bio，MB1112) or naringenin (100 mg/kg; Shanghai Yuanye Bio-Technology Co，S25634) were administered to the heterozygous pregnant females by gavage three times, at 9:00 am on gestational day 17.5, at 16:00 pm on gestational day 17.5, and at 9:00 am on gestational day 18.5, respectively. The equal amount solvents were also administrated to the control heterozygous pregnant females via the same methods. The tissues from embryos were collected 6 hours after the last administration.

**Cell experiments**

Caco-2 cells were purchased from the Cell Resource Center of Shanghai Institutes for Life Sciences, Chinese Academy of Sciences. Caco-2 cells were cultured in DMEM medium (Gibco, C11995500BT) containing 10% FBS (Gibco, 10270-106)), 10000U/ml penicillin and 10000μg/ml streptomycin (Gibco, 15140-122), in an incubator at 5% CO2, 37°C.

Caco-2 cells were seeded into 96-well plates, 5000 cells per well, and the cells were grouped according to the experimental requirements. In order to explore the appropriate concentration of Mitomycin A (MitA) on inhibiting the activation of SP1 in Caco-2 cells, the experimental group was added with MitA (0.01, 0.014, 0.02, 0.05, 0.1, 0.2 and 1μM; Meilun Bio, MB5430), and the control group was added with equal amount of DMSO; after 36h or 48h of incubation, 10μl of CCK8 reagent (Meilun Bio, MA0218-2) was added to each well, and then the cells were taken out of each well after 2h of incubation, and absorbance was measured by a microplate reader at 450nm.

In order to inhibit the activation of SP1 in Caco-2 cells, the cells were incubated with 75, 100 and 150nM of MitA, and then the cells were harvested for experiments after 36h or 48h of incubation.

To inhibit the activation of mTORC1 in Caco-2 cells, the cells were incubated with 200 μmol/ml rapamycin (VETEC, V900930), and the cells were harvested for experiments after 48h of incubation.

**Histological analysis**

The intestinal tissues were fixed in 4% paraformaldehyde at 4°C overnight. After fixation, they were dehydrated and embedded in paraffin. The 4 μm sections were stained with hematoxylin for 2 min and then with eosin for 30 s. Images were obtained using an Olympus DP74 microscope.

**Quantitative real-time polymerase chain reaction (qRT-PCR)**

RNA extraction from intestinal tissues and cells was performed using Trizol reagent (Invitrogen; Thermo Fisher Scientific), and reverse transcription was performed by PrimeScript™ RT kit (Takara Bio, Inc.) at 37°C for 15 min followed by 85°C for 5 s. The qPCR was performed using the SYBR premix Ex Taq kit (Takara Bio, Inc.) and the LightCycler 480II system (Roche, Inc.). The thermal cycling program was as follows: 95°C for 30 s; then 95°C for 5 s, 60°C for 20 s, and 65°C for 15 s, for a total of 40 cycles. Mouse or human GAPDH were used as internal reference in mouse intestinal tissues and Caco-2 cells, respectively. Primers were produced by Shanghai Sangong Biotechnology, and sequences of them were listed in Table S1.

**Western blot**

The intestinal tissues or Caco-2 cells were lysed in RIPA lysis buffer (Dalian Meilun Biotechnology Co., Ltd.) containing 2% PMSF, 2% protease inhibitor mixture, and 2% protein phosphatase inhibitor, and then centrifuged at 12,000 rpm, 4°C, for 15 min and the supernatant was harvested. Protein concentration was determined using a BCA kit (P0011, Beyotime). A 5-12% gel was selected for electrophoresis according to the molecular weight of the proteins, and equal amounts of proteins (30 μg-40 μg) were separated by SDS-PAGE, and then the separated proteins were transferred to a PVDF membrane. The PVDF membranes were blocked with 5% skimmed milk or 5% BSA for 1h at room temperature, followed by overnight incubation with primary antibody at 4 °C, and then incubated with HRP (horseradish peroxidase)-labeled secondary antibody. The resulting signals were detected using enhanced chemiluminescence reagents (Bio-Rad Laboratories, Inc; 170-5060). Primary and secondary antibodies used for protein immunoblotting were listed in Table S2.

**Immunofluorescence staining**

The immunofluorescence staining was performed as previously reported ^3^. The intestinal tissues were fixed with 4% paraformaldehyde at 4°C overnight, then sucrose gradient dehydrated, embedded in OCT compounds and sectioned. The 7 μm frozen sections were boiled in 10 mM citric acid (Merck) at pH 6.0 for 5 min, and the non-specific sites were blocked with goat serum blocking buffer (ZSGB-BIO, ZLI-9056) for 1h at room temperature. After blocking, the sections were incubated with the primary antibody overnight at 4°C, and then with the secondary antibody for 1h at room temperature. Primary antibody was rabbit anti-p-SP1 (1:200; Thermo Fisher Scientific, PA5-1004771). Immunofluorescence analysis was performed using Alex Fluor 488-labeled secondary antibody (Invitrogen). Immunofluorescence images were obtained using an Olympus confocal microscope.

**Statistical analysis**

The software GraphPad Prism 8 was used for all the graphs done in this experiment, and the statistical software was SPSS software (IBM, version 25.0). Data were expressed as mean ± standard deviation (SD). Two groups were compared using t-test, and more than two groups were analyzed using one-way ANOVA. P<0.05 was considered to be significant.

**REFERENCES**

1. Percie du Sert, N.*, et al*. The ARRIVE guidelines 2.0: updated guidelines for reporting animal research. *The Journal of physiology*. **598**, 3793-3801 (2020).
2. Yang, Y.*, et al*. Circular RNA profile in liver tissue of EpCAM knockout mice. *International journal of molecular medicine*. **44**, 1063-1077 (2019).
3. Lei, Z.*, et al*. High dose lithium chloride causes colitis through activating F4/80 positive macrophages and inhibiting expression of Pigr and Claudin-15 in the colon of mice. *Toxicology*. **457**, 152799 (2021).

**SUPPLEMENTAL FIGURES**

**
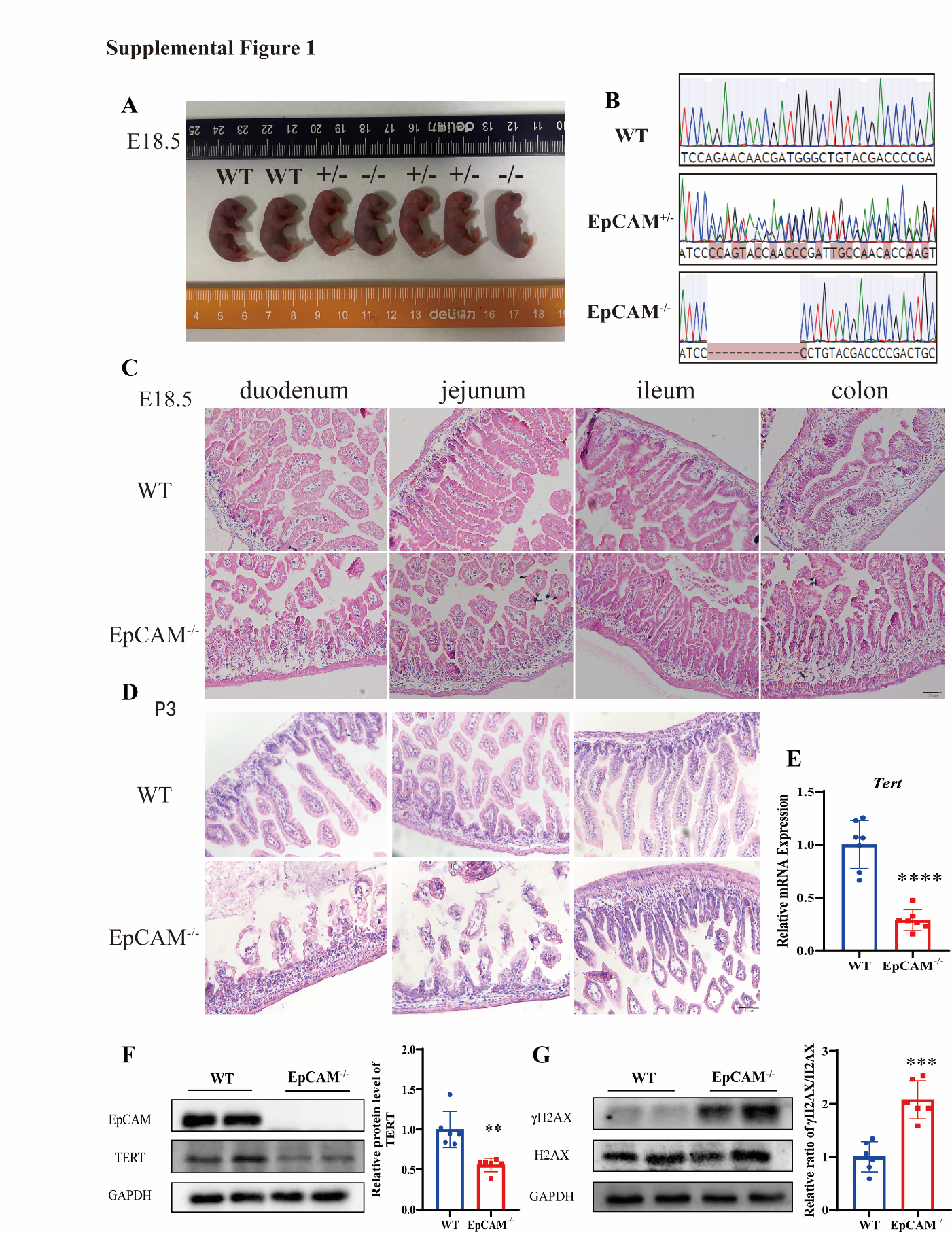
**

**Figure S1. The premature aging of intestinal epithelium in EpCAM deficient mice**

**A**. Seven E18.5 embryos of a littermate from EpCAM^+/-^ parents. The genotypes of embryos have been calibrated; **B**. The sequencing results of DNA fragments in the EpCAM gene of WT, EpCAM^+/-^ and EpCAM^-/-^ embryos respectively. **C**. Representative H&E staining images of duodenum, jejunum, ileum and colon from WT and EpCAM^–/–^ mice at E18.5 stage. Scale bar, 50 μm; **D**. Representative H&E staining images of duodenum, jejunum and ileum from WT and EpCAM^–/–^ mice at P3 stage. Scale bar, 50 μm; **E**. The relative mRNA expression levels of *Tert* in the small intestines of WT and EpCAM^-/-^ mice at E18.5 stage (n=6, ^****^P<0.0001 compared with WT group); **F**. Western blot results showed protein levels of EpCAM and TERT in the small intestines of WT and EpCAM^-/-^ mice at E18.5 stage; right panel: quantification data (n=6, ^**^P<0.01 compared with WT group); **G**. Western blot results showed protein levels of H2AX and γH2AX in the small intestines of WT and EpCAM^-/-^ mice at E18.5 stage; right panel: quantification data (n=6, ^***^P<0.001 compared with WT group). H&E, hematoxylin and eosin.


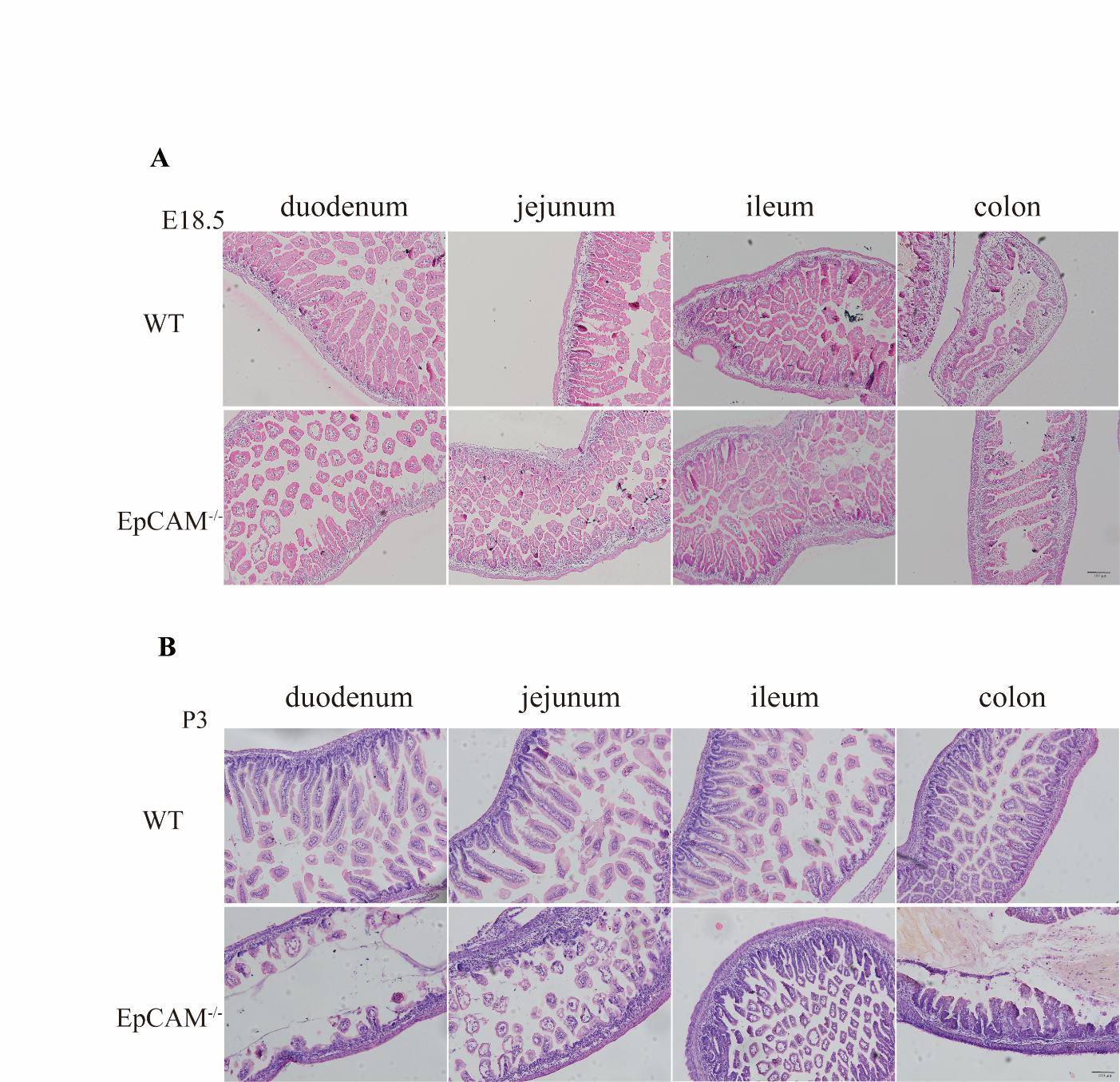


**Figure S2. The histological staining results demonstrated the intestinal failure of EpCAM^–/–^ mice shortly after birth**

**A**. Representative H&E staining images of duodenum, jejunum, ileum and colon from WT and EpCAM^–/–^ mice at E18.5 stage. Scale bar, 100 μm; **B**. Representative H&E staining images of duodenum, jejunum, ileum and colon from WT and EpCAM^–/–^ mice at P3 stage. Scale bar, 100 μm


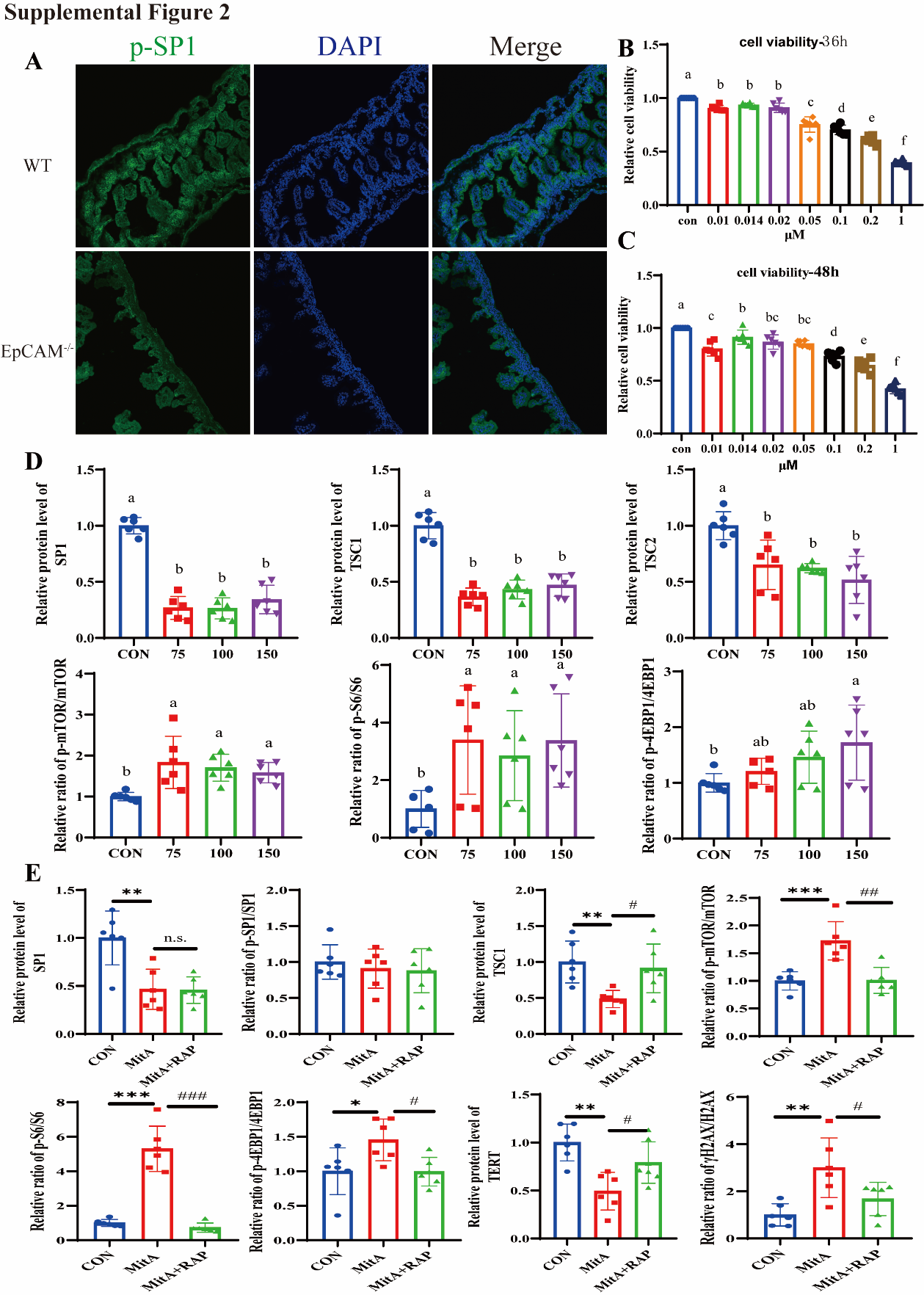


**Figure S3. EpCAM deficiency led to the reduction of SP1 activity in intestinal epithelial cells**

**A**. Immunofluorescence staining for p-SP1 in the duodenum of WT and EpCAM^-/-^ embryos at E18.5 stage. Scale bar, 50 μm; **B**. Determination of CCK-8 toxicity in Caco-2 cells treated with different concentrations of MitA for 36h, respectively (n=6, a, b, c, d, e, f indicate significant differences between groups labeling different letters, and the order of the letters a, b, c, d, e, f also refers to the relative quantity from high to low); **C**. CCK-8 toxicity assay of Caco-2 cells treated with different concentrations of MitA for 48h respectively (n=6, a, b, c, d, e, f indicate significant differences between groups labeling different letters, and the order of the letters a, b, c, d, e, f also refers to the relative quantity from high to low); **D**. The quantification data of western blot results for Figures 1D-1E; (n=6, a, b indicate significant differences between groups labeling different letters, and the order of the letters a, b also refers to the relative quantity from high to low); **E**. The quantification data of western blot results for Figures 1I, 1J and 1L (n=6, ^*^P<0.05, ^**^P<0.01 and ^***^P<0.001 compared with CON group, ^#^P<0.05 and ^####^P<0.001 compared with MitA group, respectively; n.s. indicates non-significant statistical differences). CON, Control; 75, 100 and 150, concentrations of 75 nM, 100 nM and 150 nM of MitA in the Caco-2 cell cultures, respectively; MitA, Mithramycin A; RAP, Rapamycin.


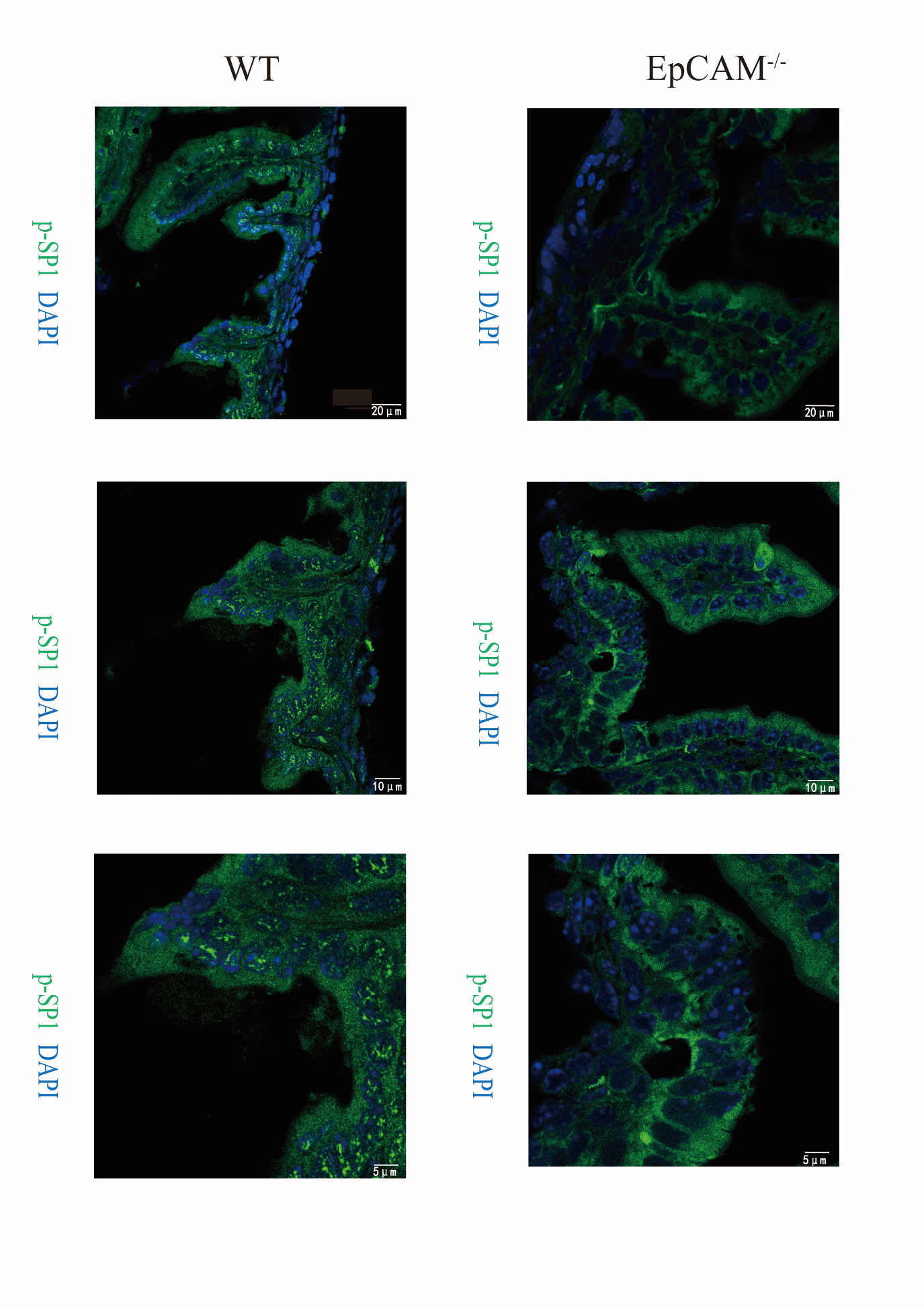


**Figure S4. The immunofluorescence staining results demonstrated the reduction of p-SP1 in the intestines of EpCAM^-/-^ pups**

Immunofluorescence staining for p-SP1 in the duodenum of WT (**A**, **C**, **E**) and EpCAM^-/-^ (**B**, **D**, **F**) pups at P3 stage. **A** and **B**, Scale bar, 20 μm; **C** and **D**, Scale bar, 10 μm; **E** and **F**, Scale bar, 5 μm.


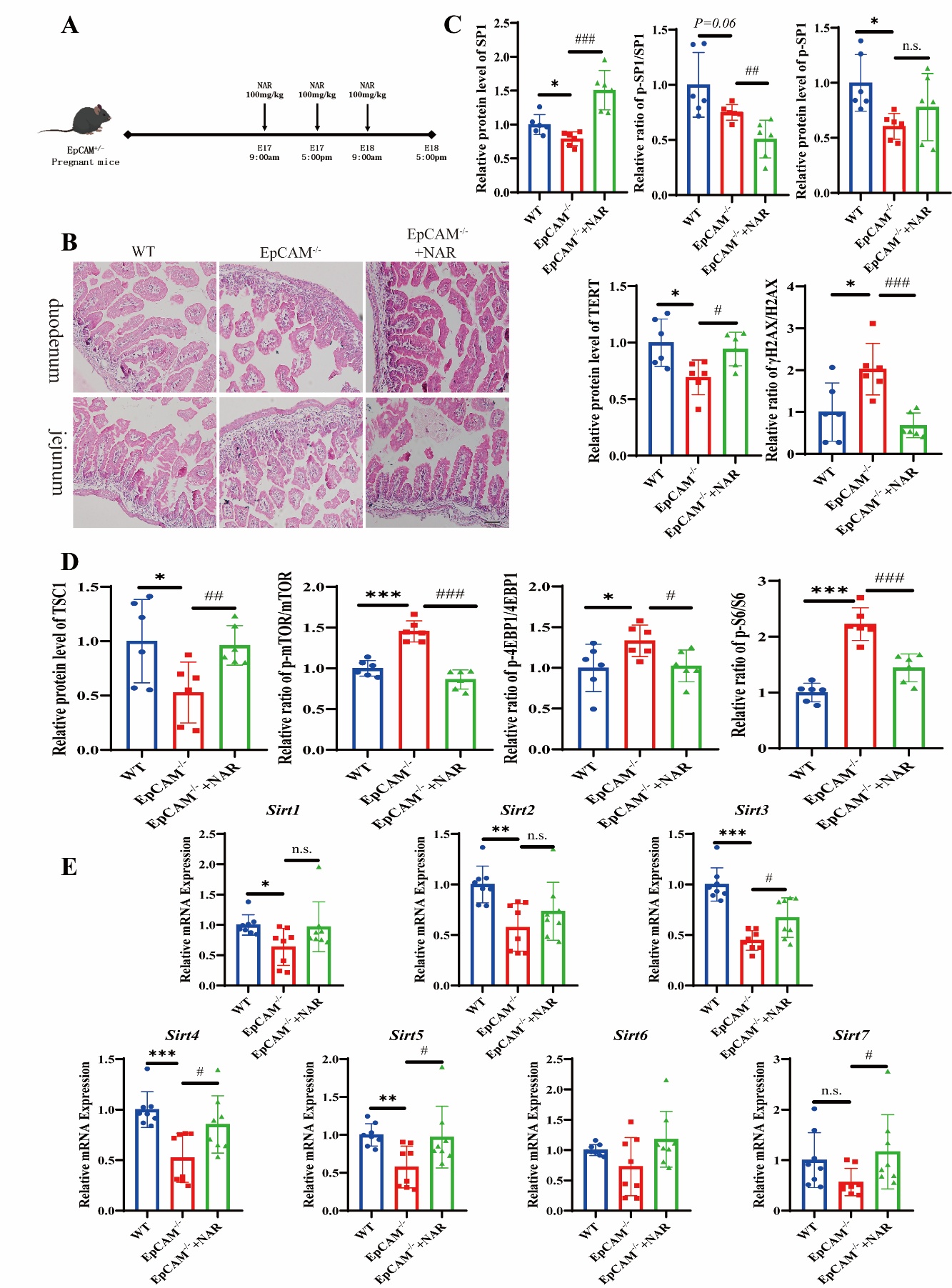


**Figure S5. Naringenin effectively rescued the premature aging of intestinal epithelial cells in the EpCAM deficient mice**

**A**. Pattern diagram illustrating the process of naringenin administration and sampling in mice; **B**. H&E staining of duodenal and jejunum tissues from mice of WT, EpCAM^-/-^ and EpCAM^-/-^ +NAR groups at E18.5 stage. Scale bar, 50 μm; **C-D**. The quantification data of western blot results for Figures 2B-2F (n=6, ^*^P<0.05 and ^***^P<0.001 compared with WT group, ^#^P<0.05, ^##^P<0.01, and ^###^P< 0.001 compared with EpCAM^-/-^ group; n.s. indicates non-significant statistical differences); **E**. The relative mRNA expression levels of *Sirt1-7* in the small intestinal tissues of mice from WT, EpCAM^-/-^, and EpCAM^-/-^ + NAR groups at E18.5 stage (n=8, ^*^P<0.05, ^**^P<0.01 and ^***^P< 0.001 compared with WT group, ^#^P<0.05 compared with EpCAM^-/-^ group; n.s. indicates non-significant statistical differences).


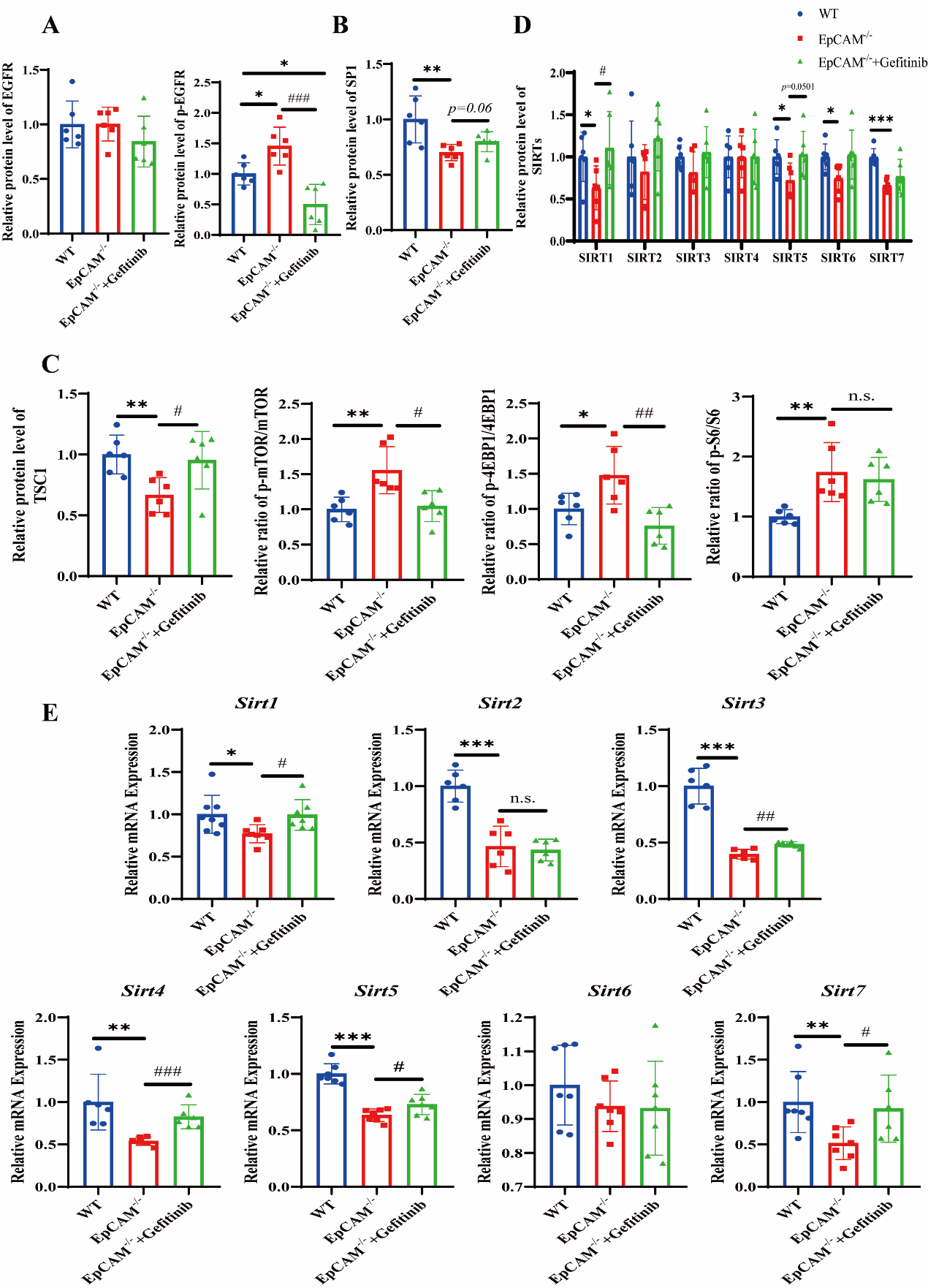


**Figure S6. The administration of gefitinib could effectively improve the hyperactivation of mTORC1 pathway and rescue the downregulations of Sirt family members in the intestinal epithelium of EpCAM deficient mice**

**A**. The quantification data for EGFR and p-EGFR in Figure 3A (n=6); **B**. The quantification data for SP1 in Figure 4B (n=6, ^**^P<0.01 compared with WT group); **C**. The quantification data for TSC1, p-mTOR/mTOR, p-4EBP1/4EBP1 and p-S6/S6 in Figure 4D (n=6, ^*^P<0.05 and ^**^P<0.01 compared with WT group, ^#^P<0.05 and ^##^P<0.01 compared with EpCAM^-/-^ group; n.s. indicates non-significant statistical differences); **D**. The quantification data of western blot results in Figure 4E (n=6, ^*^P<0.05 and ^***^P<0.001 compared with the WT group, ^#^P<0.05 compared with the EpCAM^-/-^ group); **E**. The relative mRNA expression levels of *Sirt1*, *Sirt2*, *Sirt3*, *Sirt4*, *Sirt5*, *Sirt6*, and *Sirt7* in the small intestines of mice from WT, EpCAM^-/-^ and EpCAM^-/-^ + Gefitinib groups at E18.5 stage (n=7, ^*^P<0.05, ^**^P< 0.01 and ^***^P< 0.001 compared with the WT group, ^#^P<0.05, ^##^P<0.01 and ^####^P<0.001 compared with EpCAM^-/-^ group; n.s. indicates non-significant statistical differences).


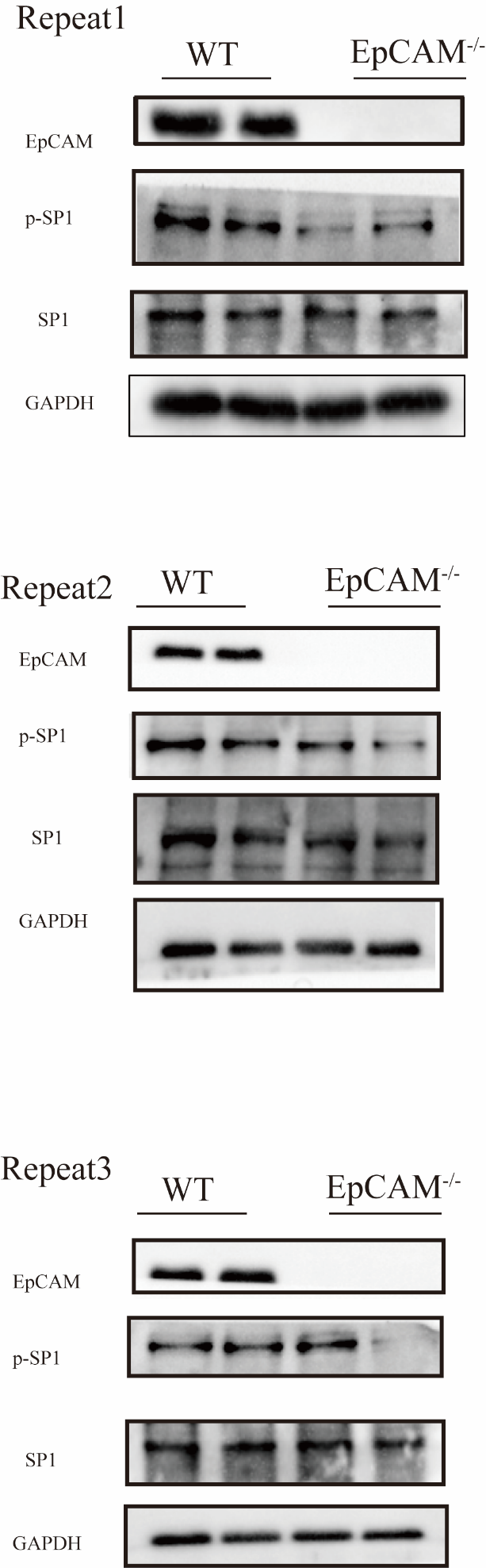


**Figure S7. The three repeats of the original western blots related to Figure 1B**


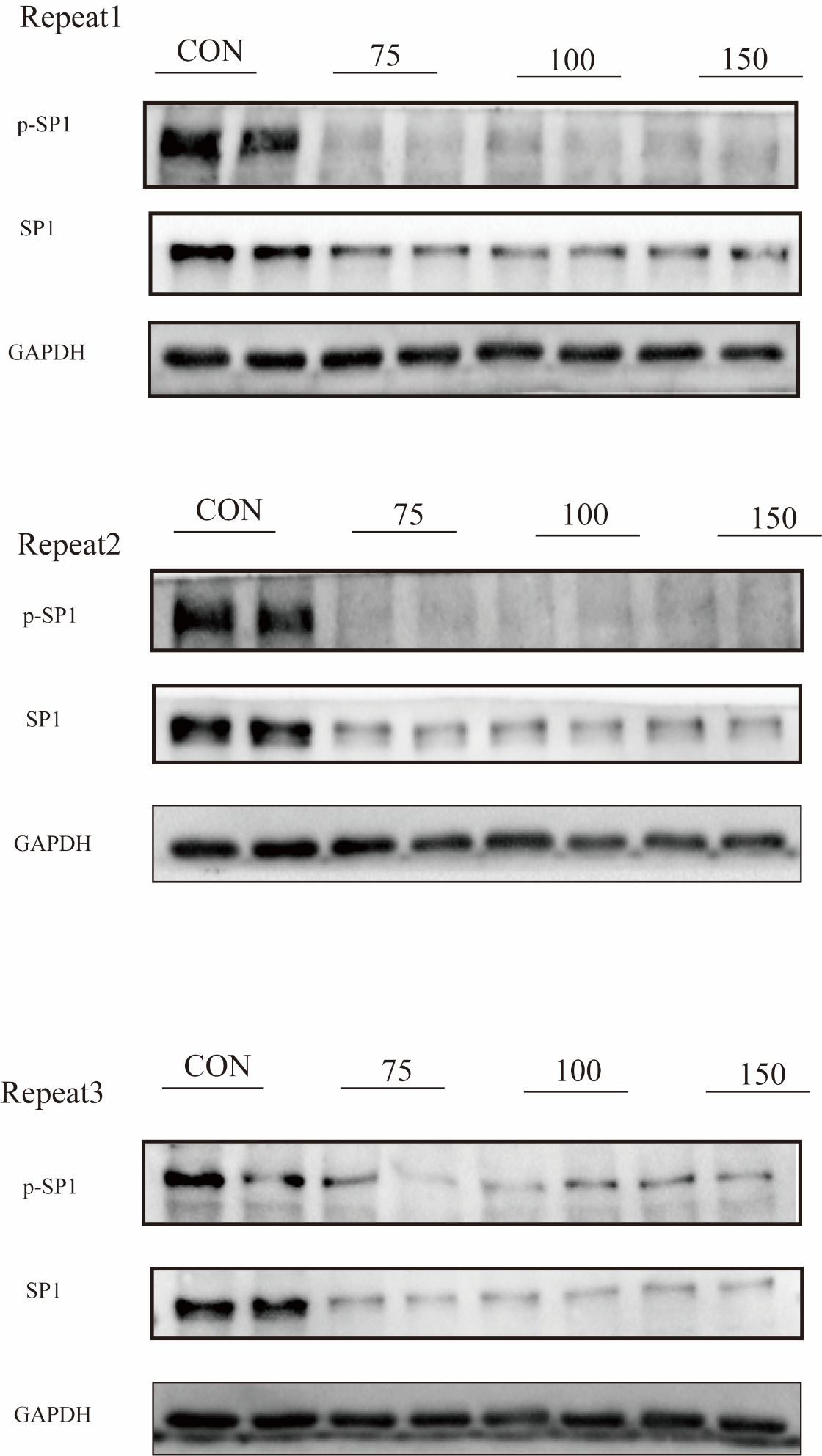


**Figure S8. The three repeats of the original western blots related to Figure 1D**


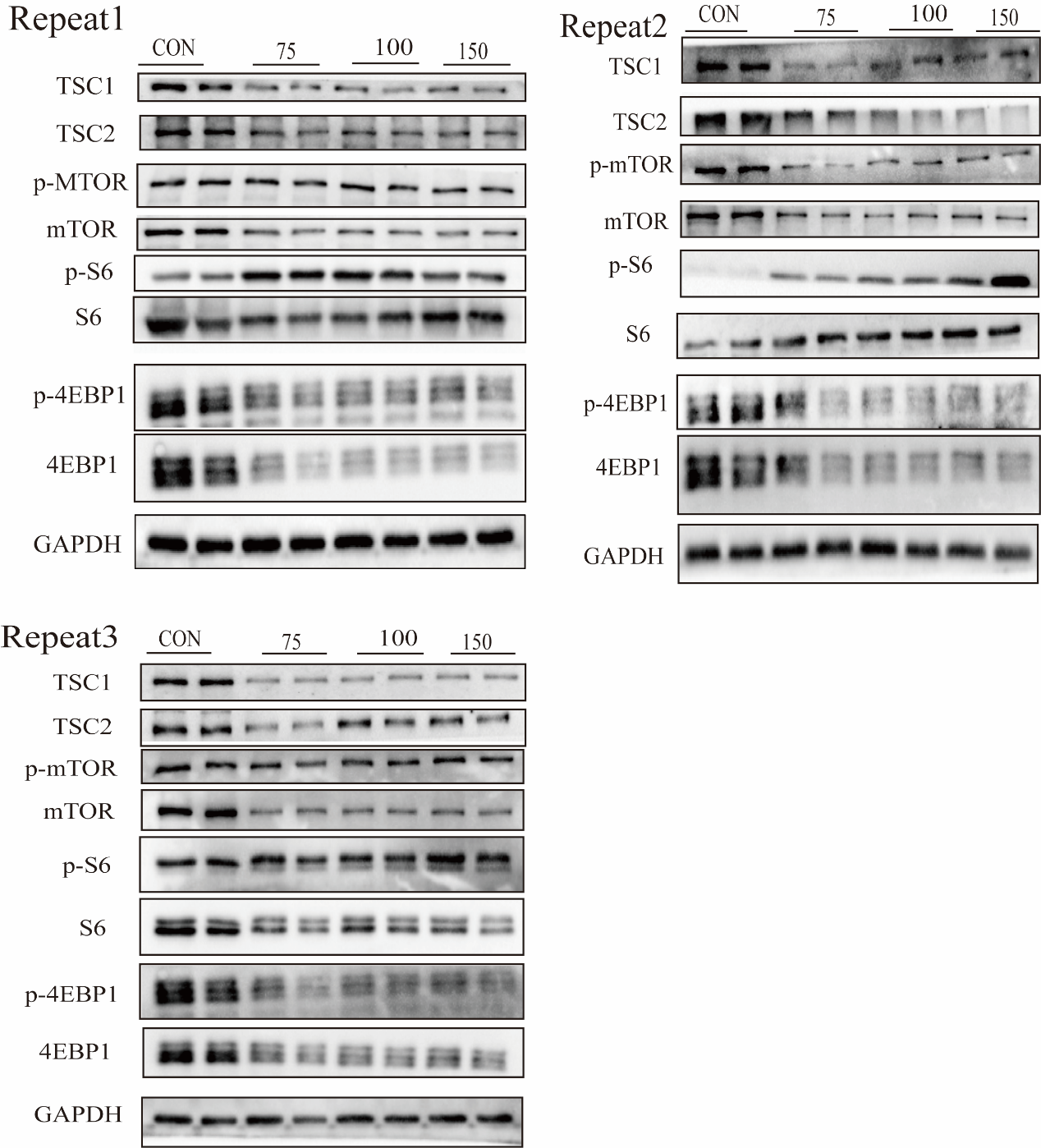


**Figure S9. The three repeats of the original western blots related to Figure 1E**


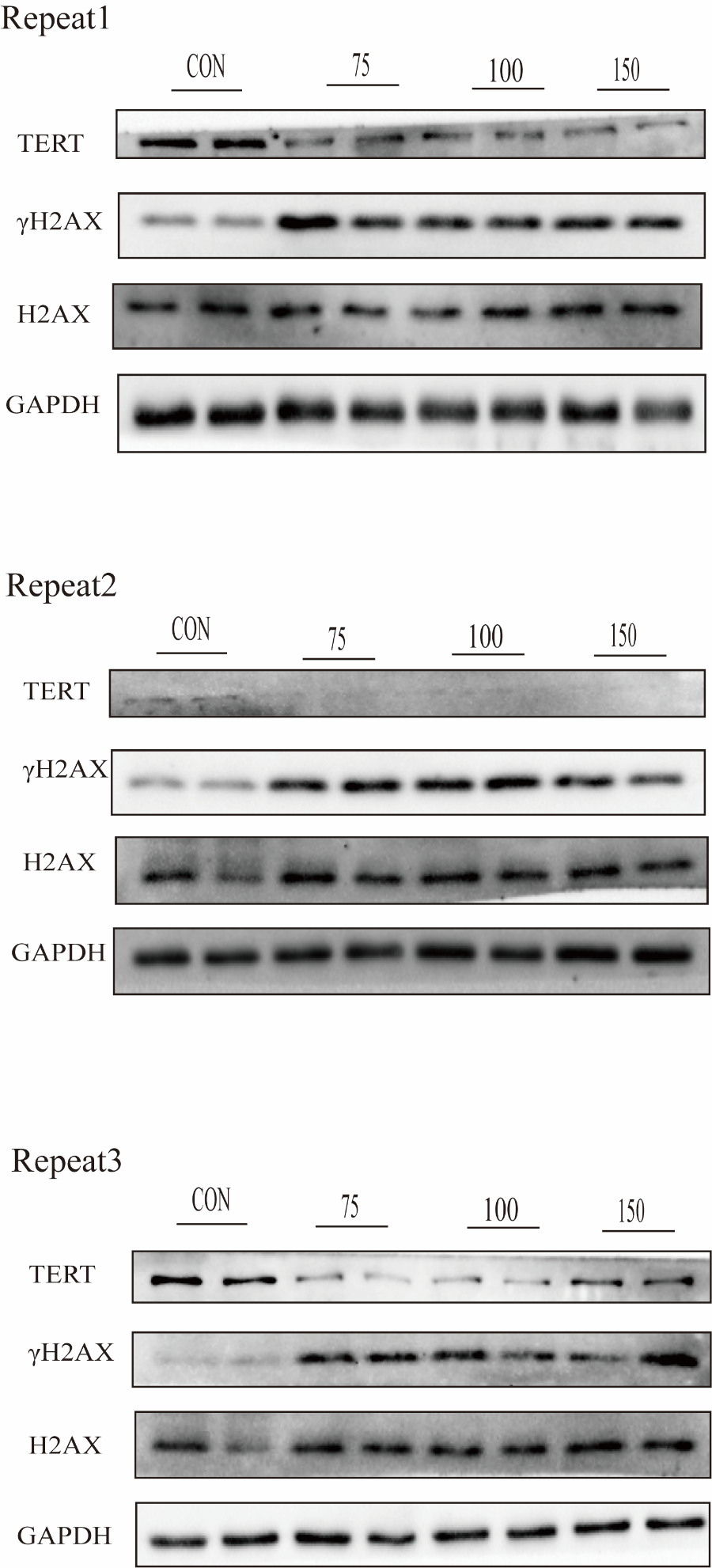


**Figure S10. The three repeats of the original western blots related to Figure 1F**


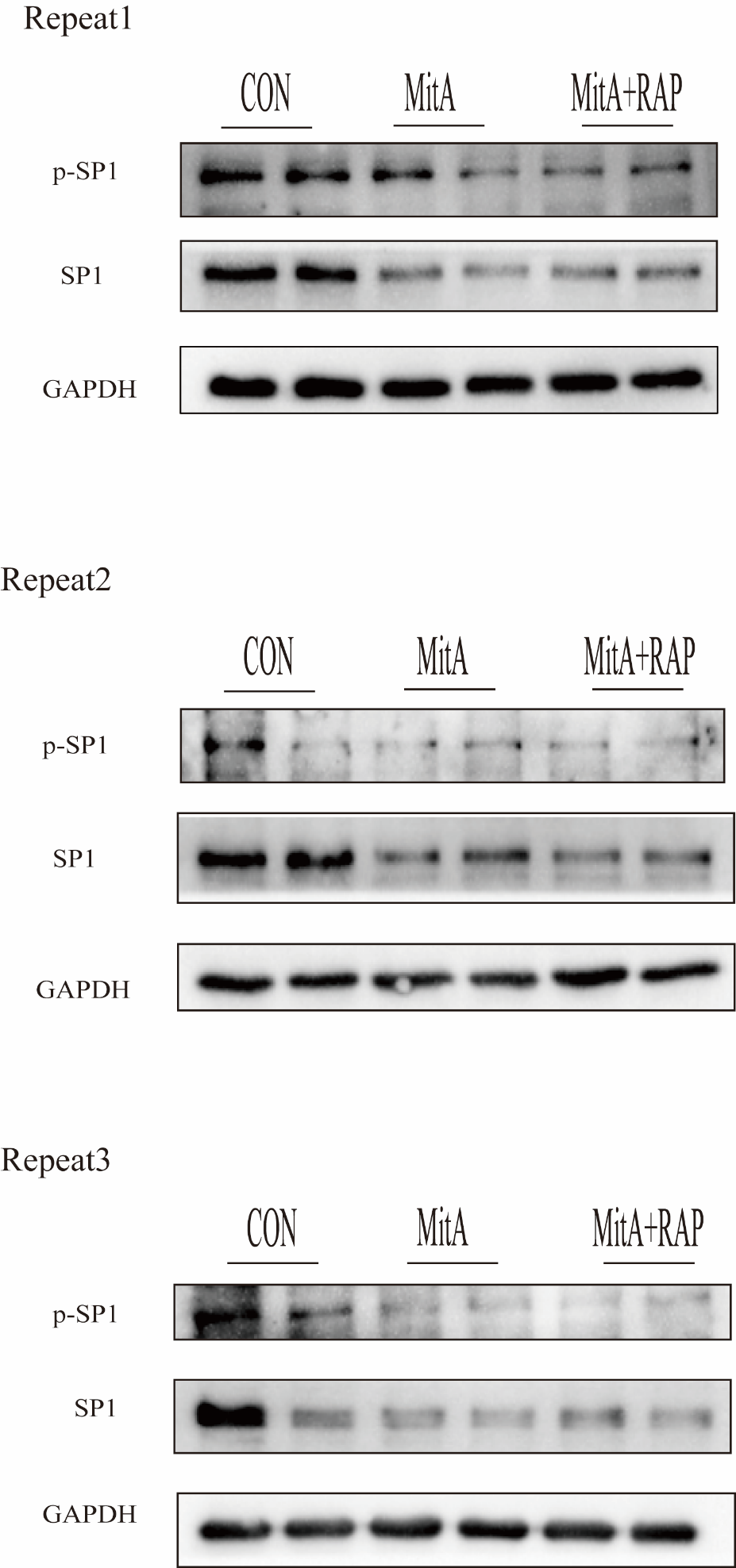


**Figure S11. The three repeats of the original western blots related to Figure 1I**


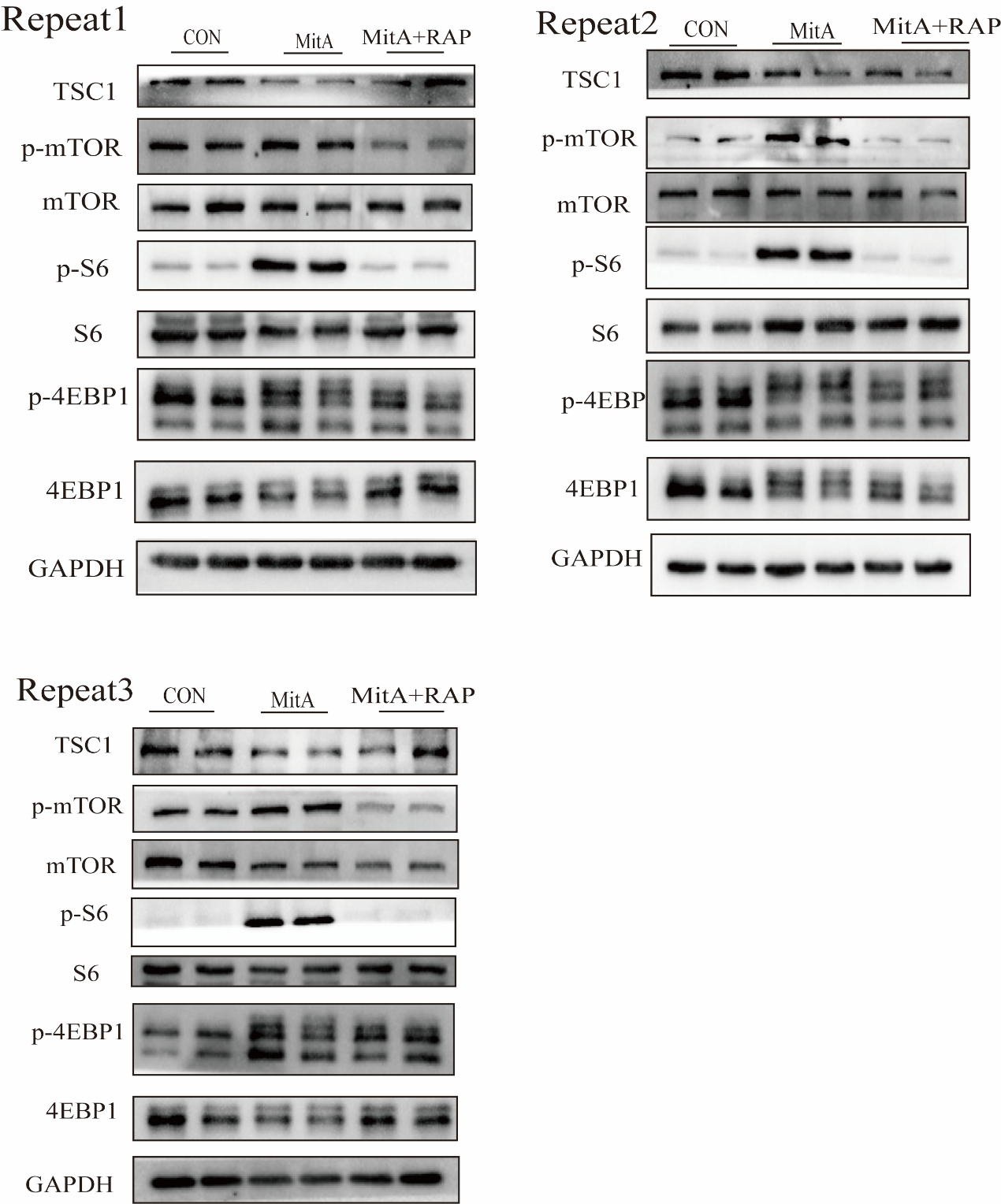


**Figure S12. The three repeats of the original western blots related to Figure 1J**


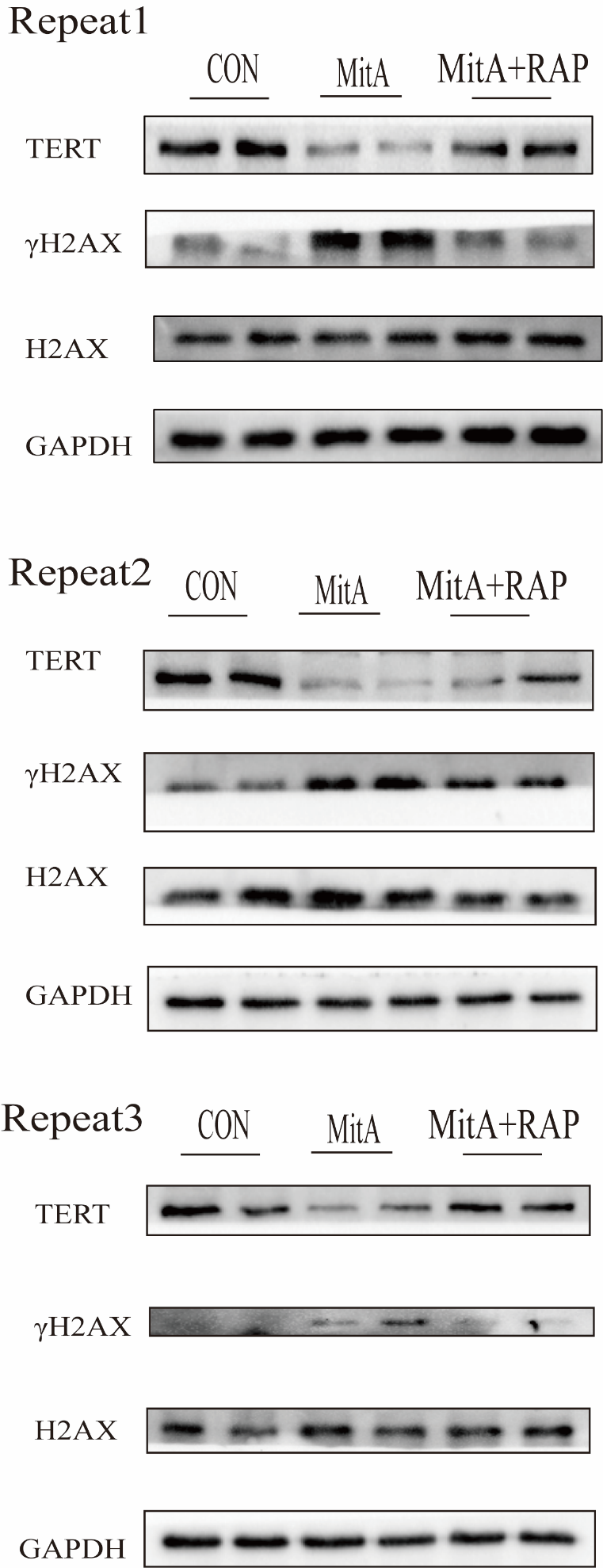


**Figure S13. The three repeats of the original western blots related to Figure 1L**


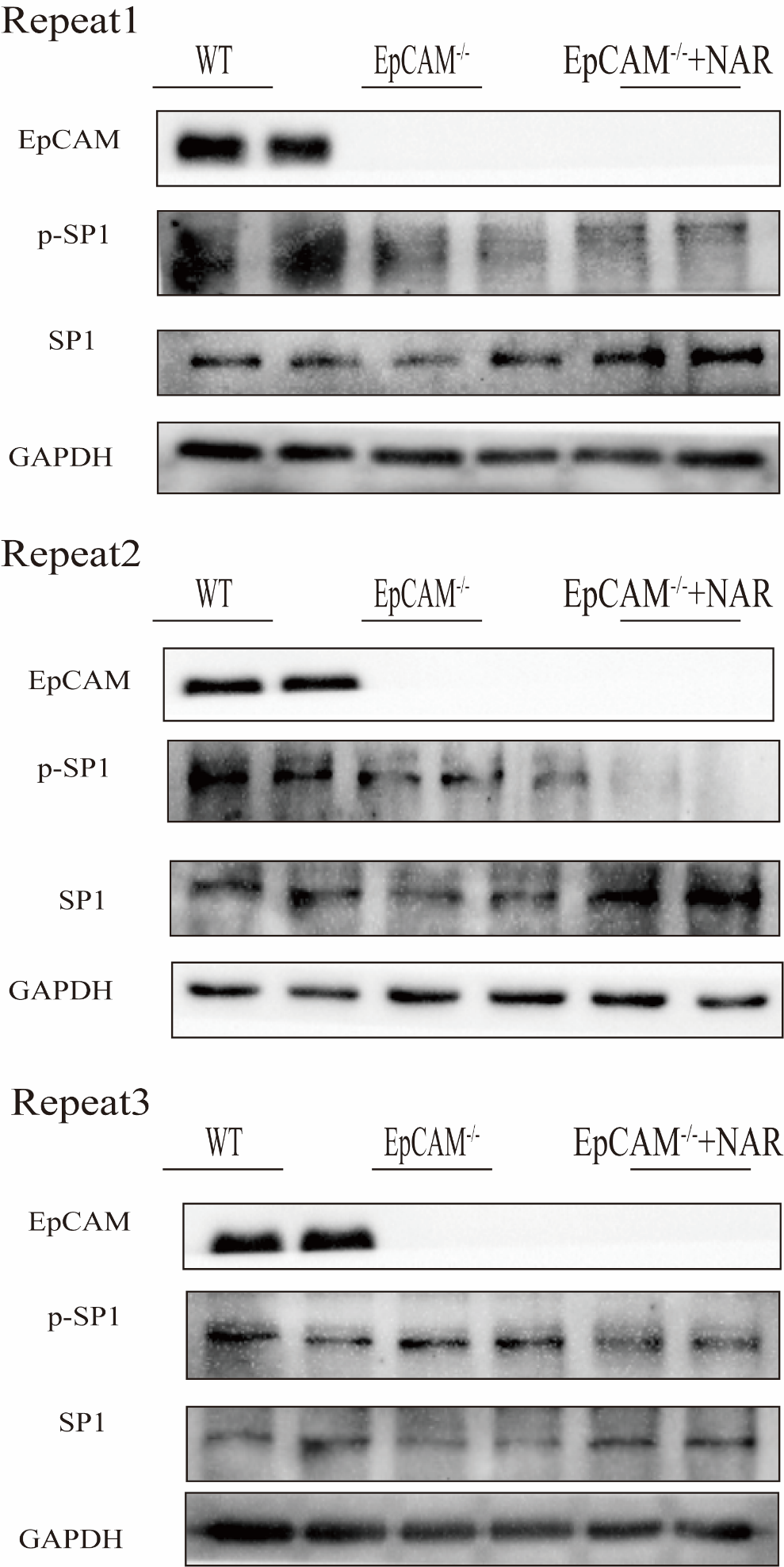


**Figure S14. The three repeats of the original western blots related to Figure 2B**


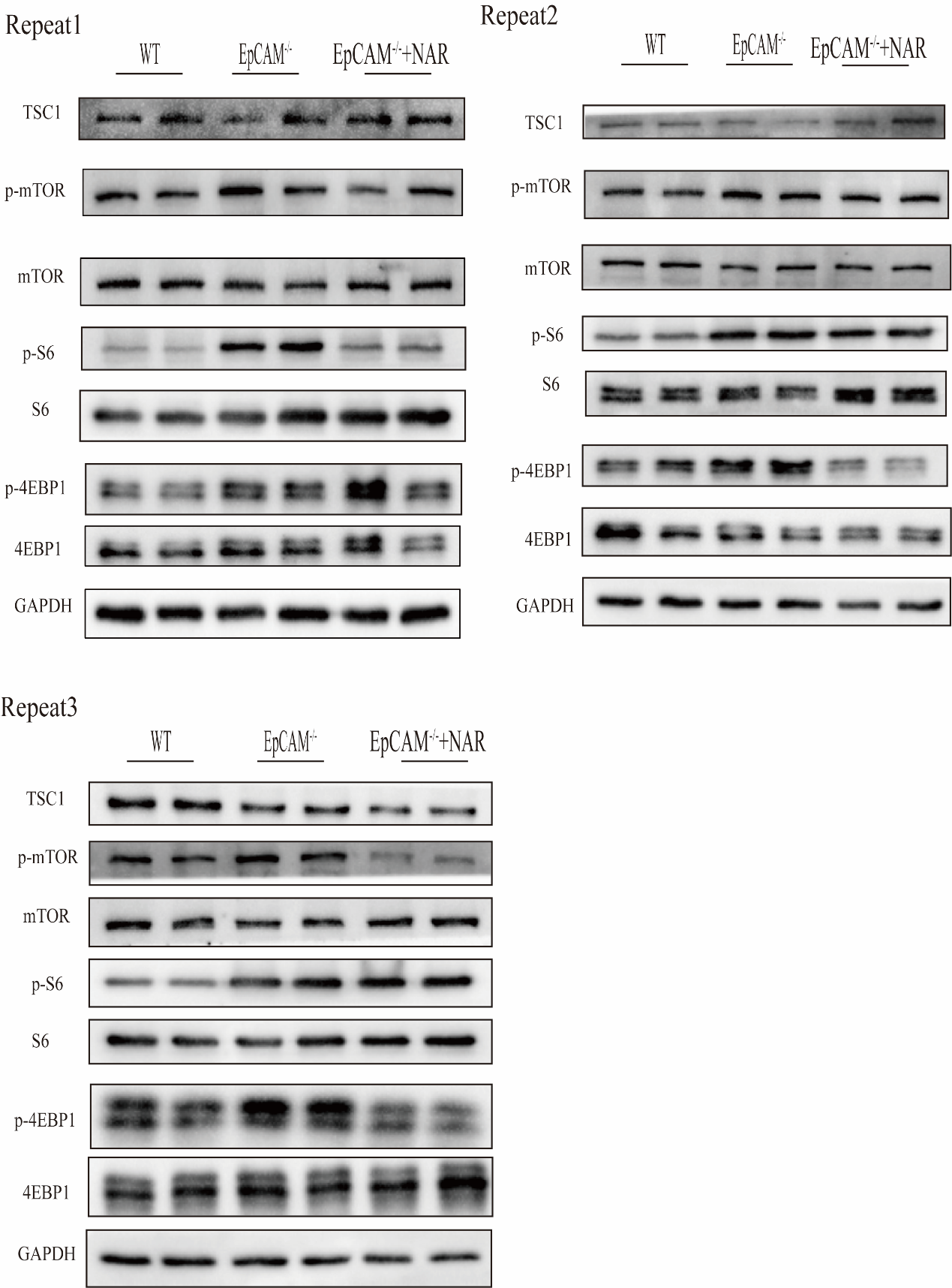


**Figure S15. The three repeats of the original western blots related to Figure 2C**


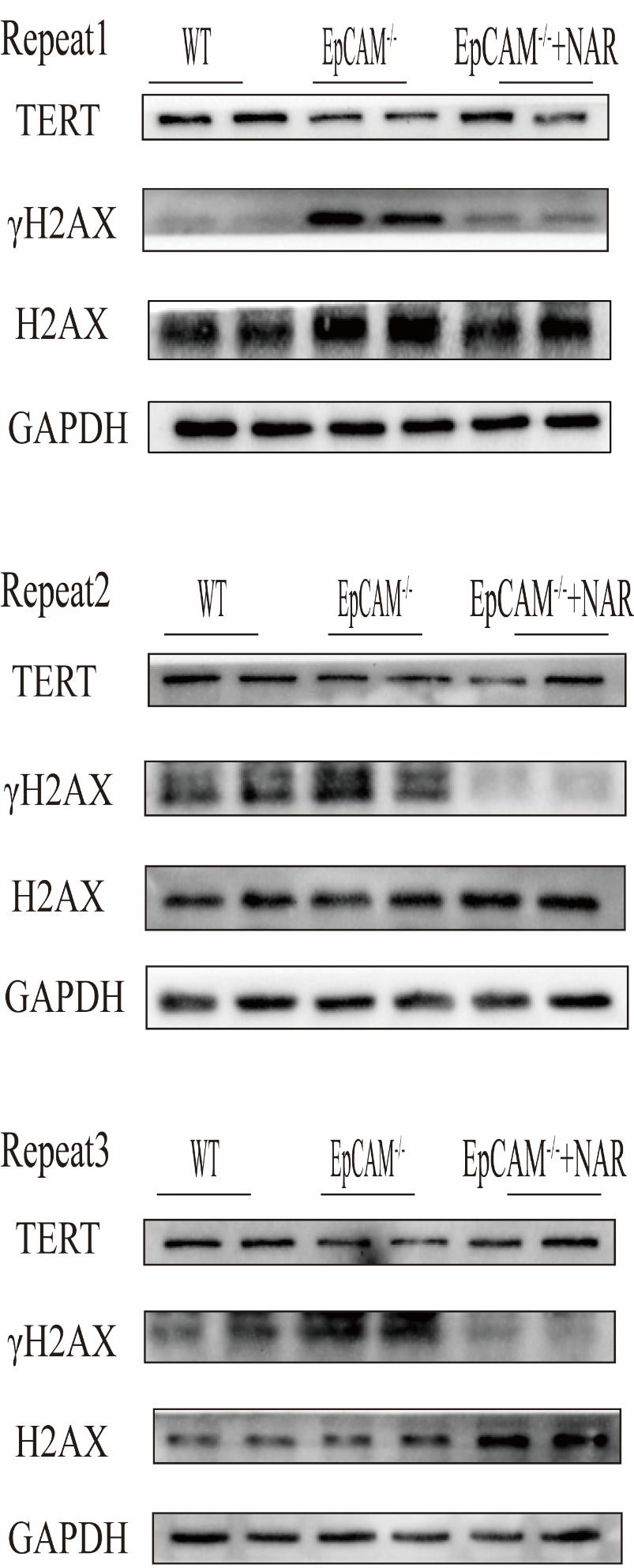


**Figure S16. The three repeats of the original western blots related to Figure 2F**


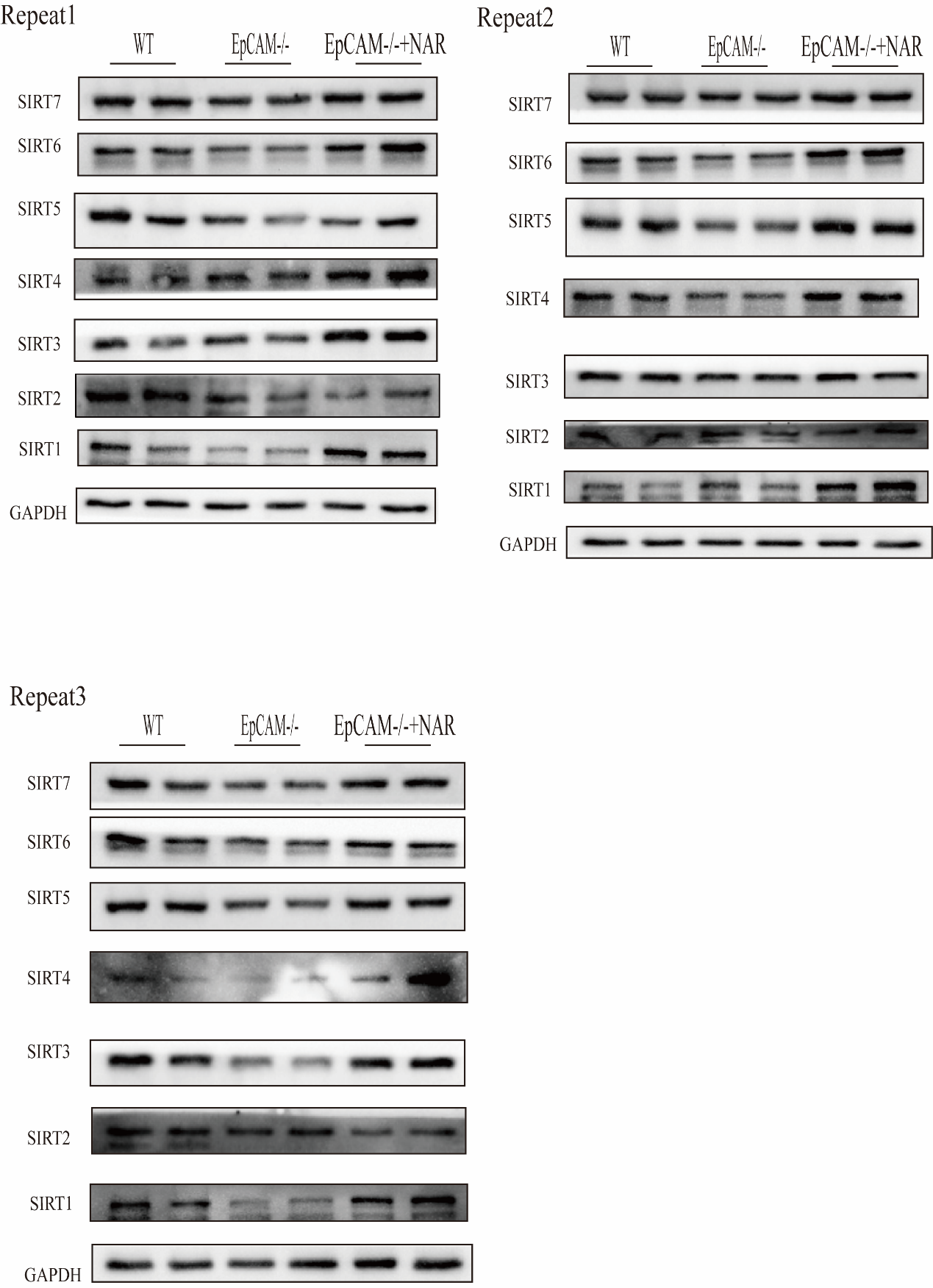


**Figure S17. The three repeats of the original western blots related to Figure 2G**


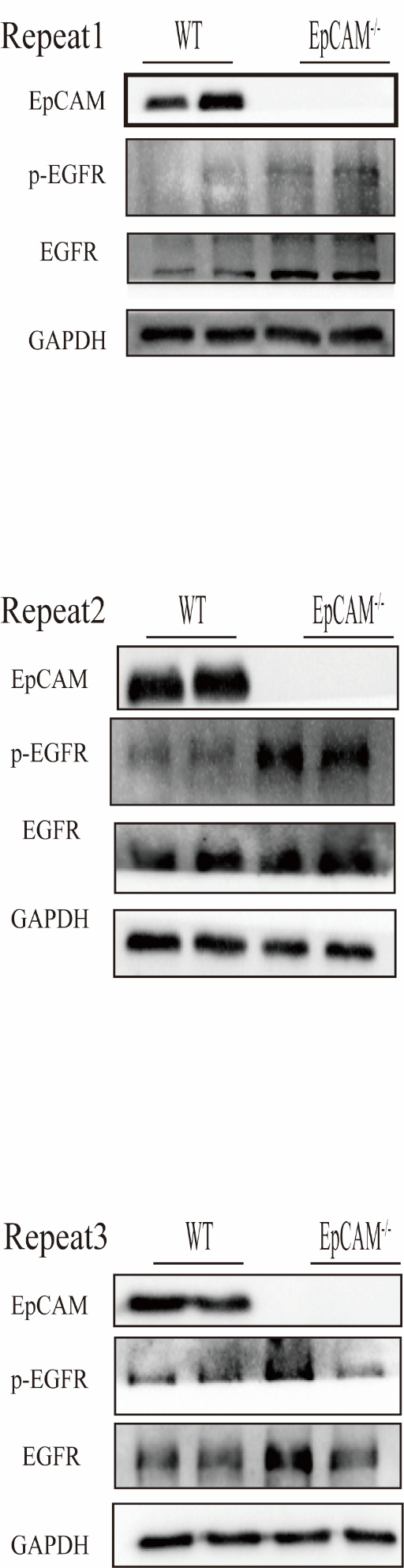


**Figure S18. The three repeats of the original western blots related to Figure 3A**


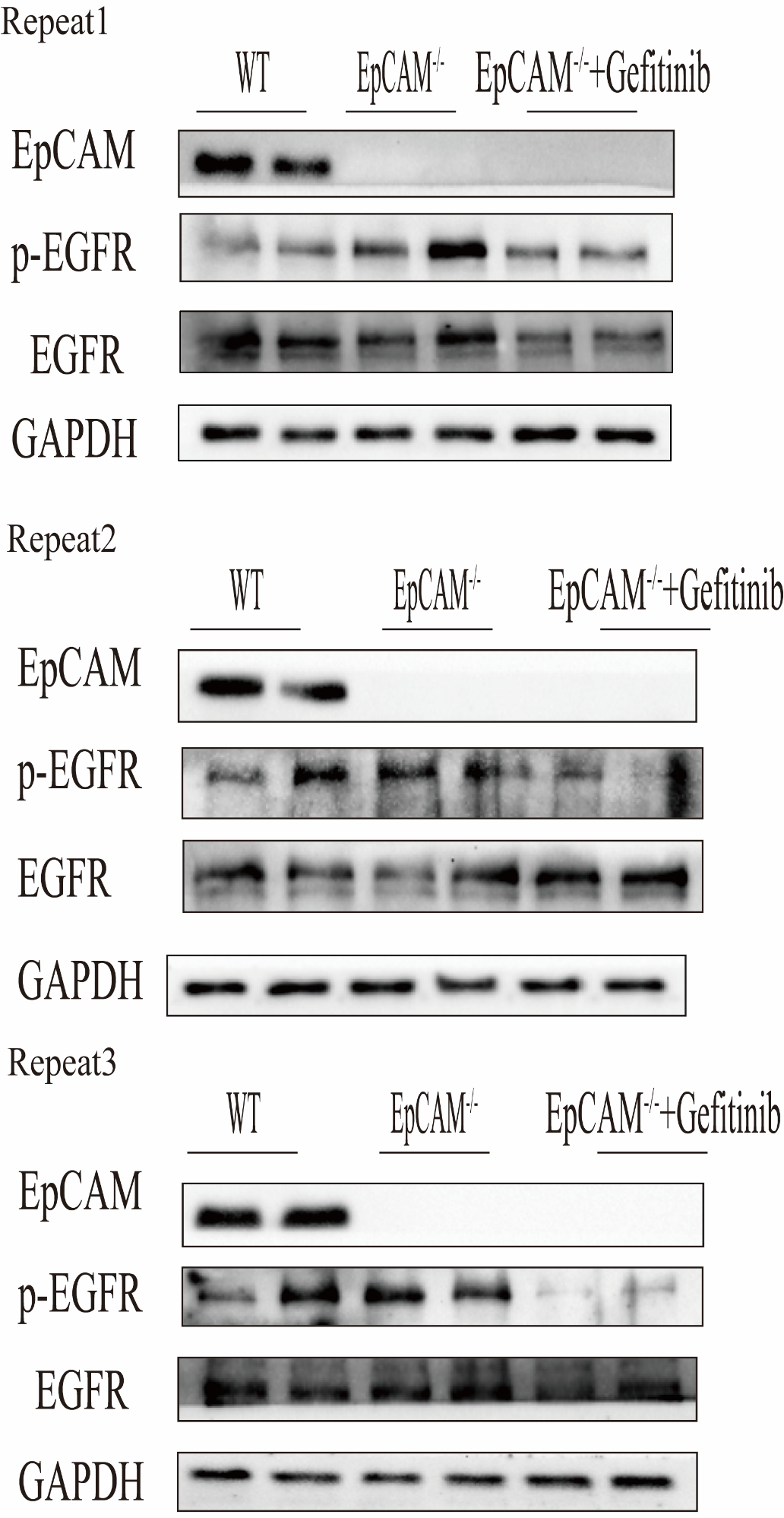


**Figure S19. The three repeats of the original western blots related to Figure 3C**


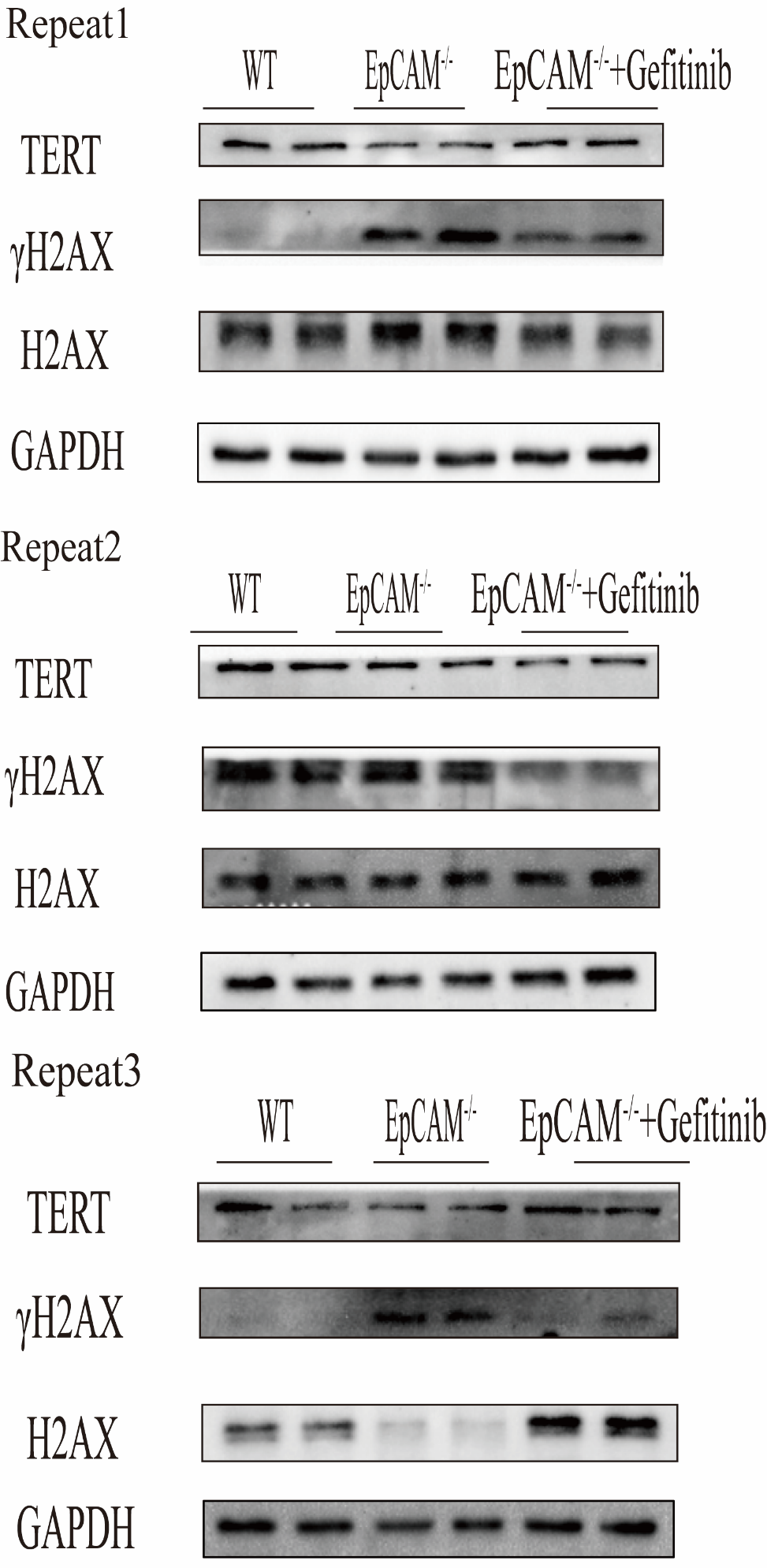


**Figure S20. The three repeats of the original western blots related to Figure 3E**


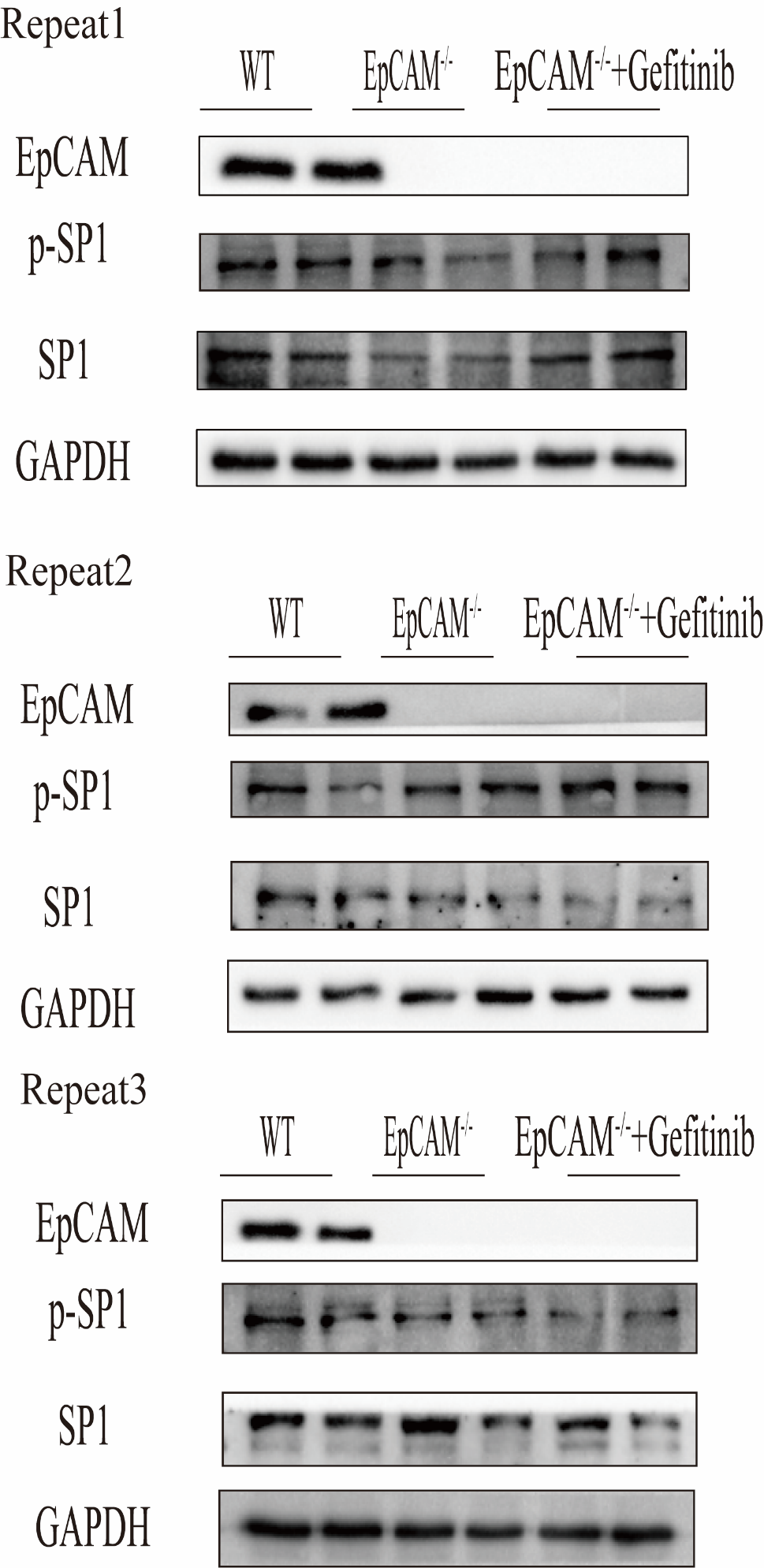


**Figure S21. The three repeats of the original western blots related to Figure 4B**


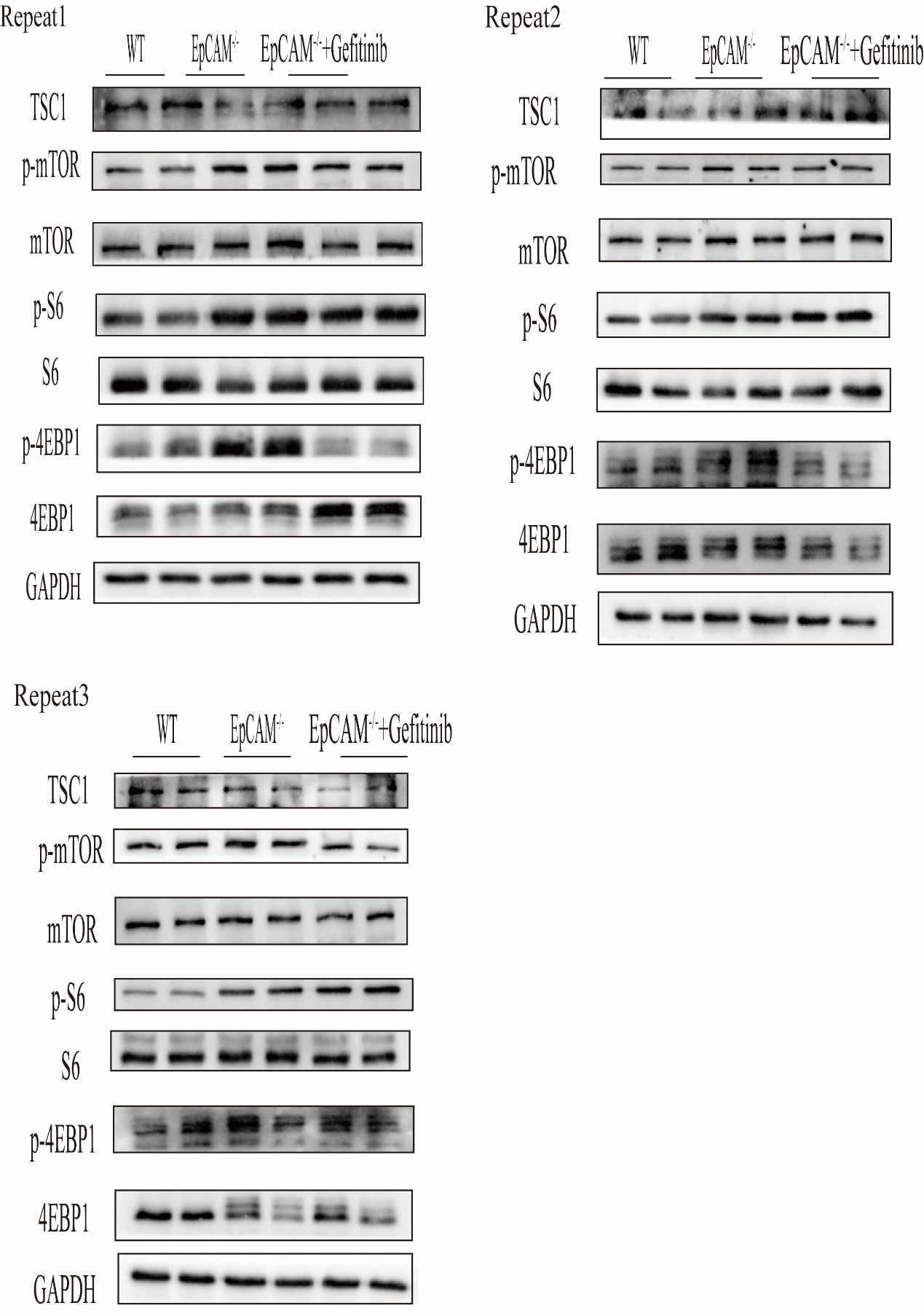


**Figure S22. The three repeats of the original western blots related to Figure 4D**


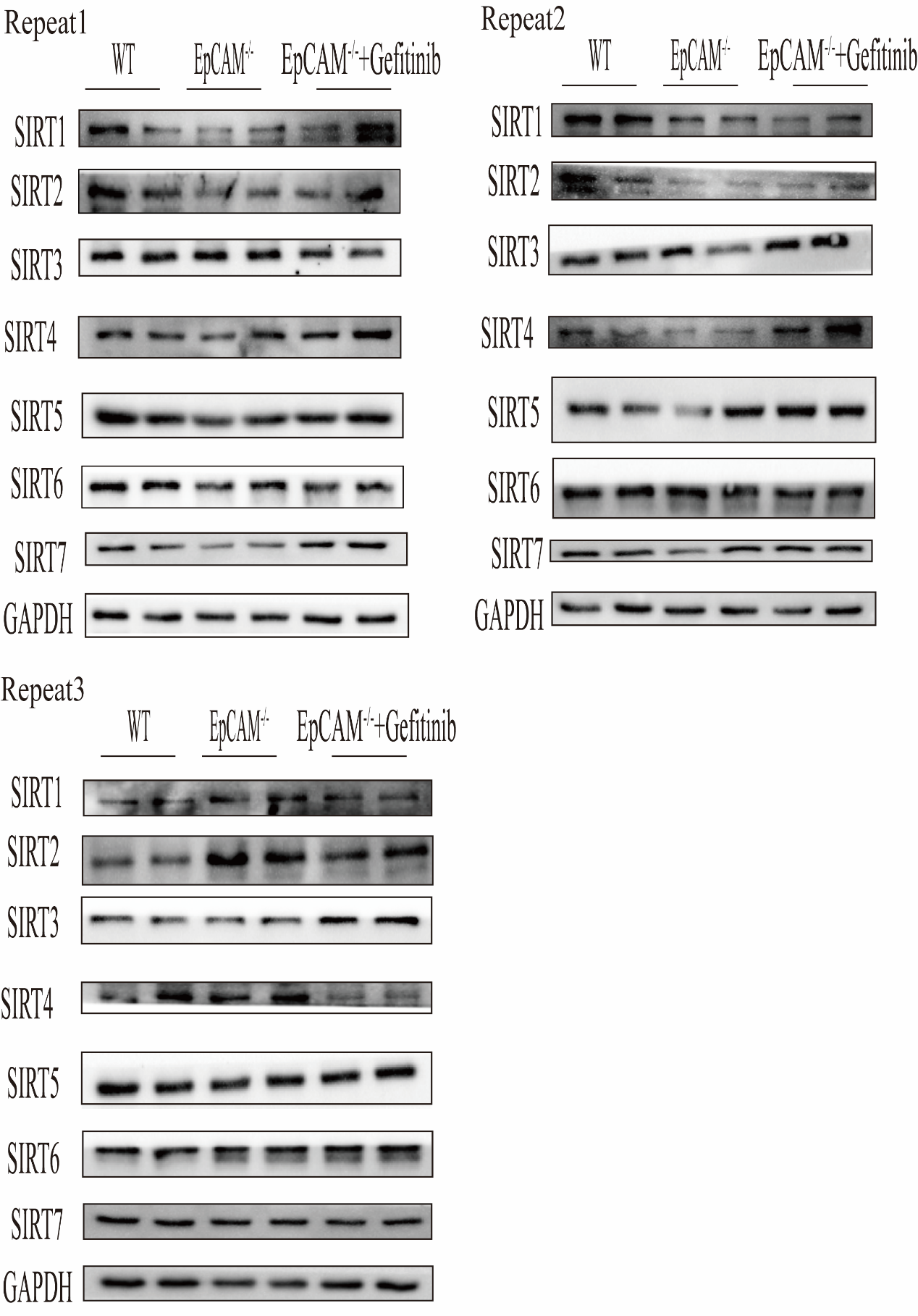


**Figure S23. The three repeats of the original western blots related to Figure 4E**


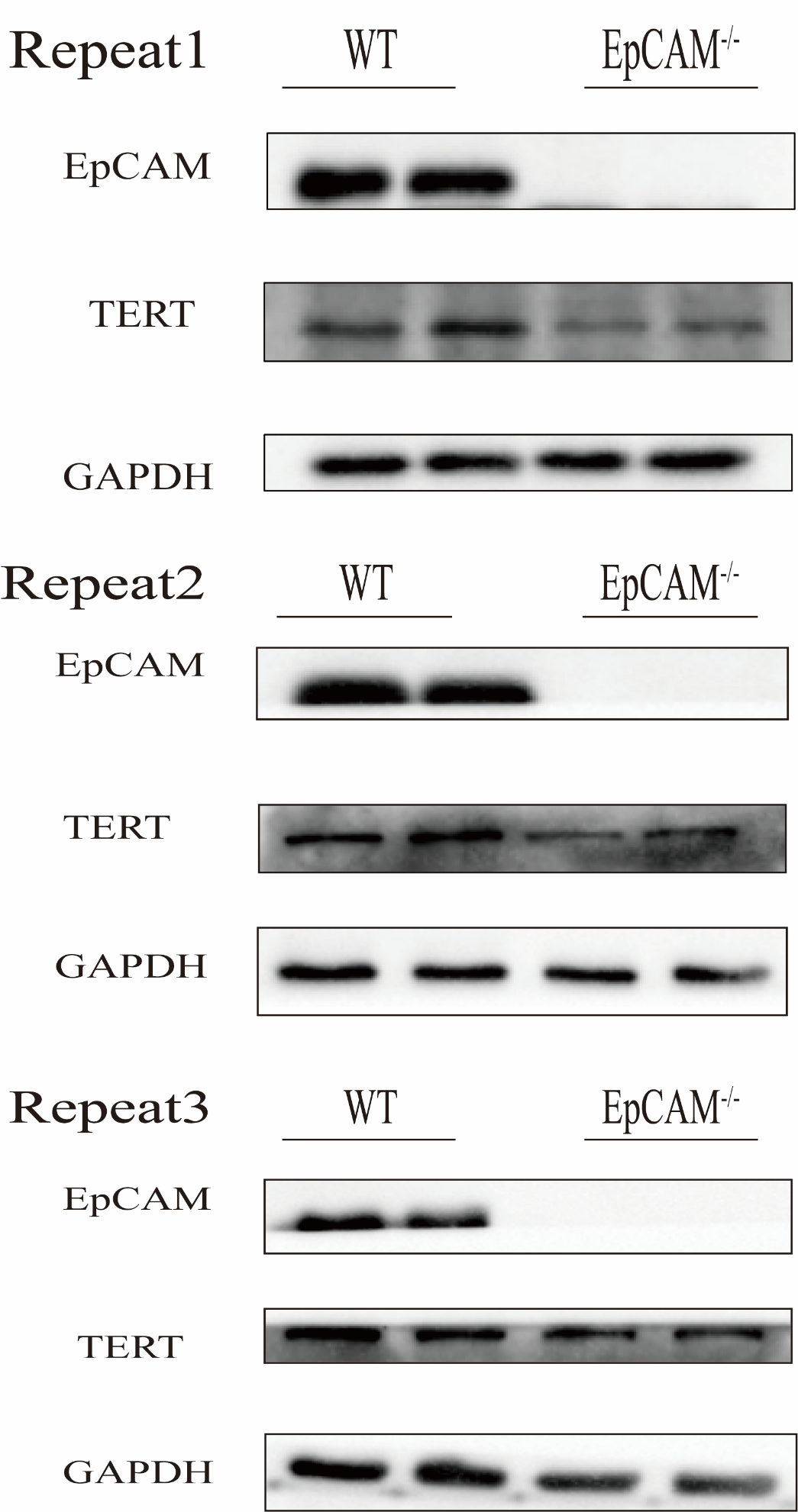


**Figure S24. The three repeats of the original western blots related to Figure S1F**


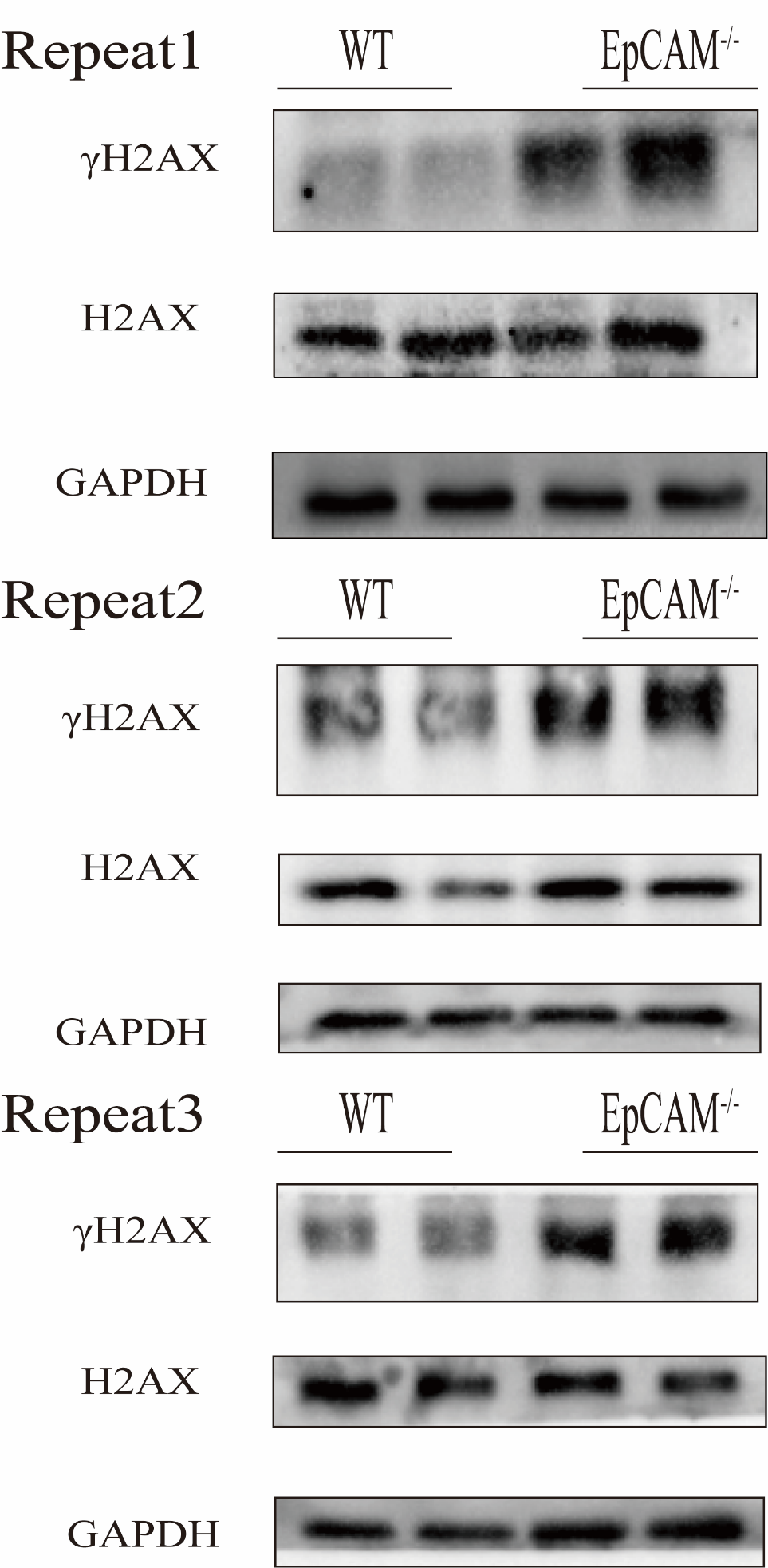


**Figure S25. The three repeats of the original western blots related to Figure S1G**

**SUPPLEMENTAL TABLES DESCRIPTION**

**Table S1. Sequences of primers used for qPCR**

| OligomerName | | | Forward primer | Reverse primer |
| --- | --- | --- | --- | --- |
| EpCAM M | | CCTGAGAGTGAACGGAGAGC | | CACAATGACAGCGATGATCC |
| Tert M | AGAGTGCCAATATGTCAGACTC | | | CGAAGAAAACCATATACCTGCC |
| Sirt1 M | CGCTGTGGCAGATTGTTATTAA | | | TTGATCTGAAGTCAGGAATCCC |
| Sirt2 M | CAGCTACTTCAAGAAACATCCG | | | TATTCTTTTCTGCAGGAGGTGT |
| Sirt3 M | TCTATACACAGAACATCGACGG | | | GCATGTAGCTGTTACAAAGGTC |
| Sirt4 M | CATCCAGCACATTGATTTCGTC | | | GTTGGTGAGAGGAGAATTGAGG |
| Sirt5 M | CGATTCATTTCCCAGTTGTGTT | | | CATATTTGAACTTGGACGAGCC |
| Sirt6 M | CCCAAGTGTAAGACGCAGTA | | | GTCCAGAATGGTGTCTCTCAG |
| Sirt7 M | GTGTGTGATGACCCGGAGG | | | TAATCTGGGCTTTGGCTGGAAG |
| EGFR M | GGACTGTGTCTCCTGCCAGAAT | | | GGCAGACATTCTGGATGGCACT |
| Sp1 M | CTCCAGACCATTAACCTCAGTGC | | | CACCACCAGATCCATGAAGACC |
| Tsc1 M | CTCTGGAGGAACACAATGCAGC | | | GGTGTCACGTTGTTCCTGAAGC |
| Tsc2 M | GAGCAGTATGCCAGCGTGTTTG | | | GCACCTGATGAACCACATGGCT |
| Gapdh M | CATCACTGCCACCCAGAAGACTG | | | ATGCCAGTGAGCTTCCCGTTCAG |
| Gapdh H | GTCTCCTCTGACTTCAACAGCG | | | ACCACCCTGTTGCTGTAGCCAA |
| Tsc1 H | CTGGACAGACTGATACAGCAGG | | | TGCGGATCTCATCTGAAGGAGG |
| Tsc2 H | GCACCTCTACAGGAACTTTGCC | | | GCACCTGATGAACCACATGGCT |
| Sp1 H | ACGCTTCACACGTTCGGATGAG | | | TGACAGGTGGTCACTCCTCATG |
| Tert H | CAAGTTGCAAAGCATTGGAATC | | | ACGTAGTCCATGTTCACAATCG |
| Sirt1 H | TATACCCAGAACATAGACACGC | | | CTCTGGTTTCATGATAGCAAGC |
| Sirt2 H | GACTCAGATTCAGACTCTGAGG | | | GAGCGTCTGGGAGAATAAGTTC |
| Sirt3 H | CCCAGTGGCATTCCAGACTT | | | AAGGGCTTGGGGTTGTGAAA |
| Sirt4 H | CATCCAGCATGGTGATTTTGTC | | | CATCCAGCACATTGATTTCGTC |
| Sirt5 H | AGATTGTCCCAAGTCGATTGAT | | | CCATTTTCAGGCAAATCTGGTT |
| Sirt6 H | CTCTGGCATCCCCGACTTC | | | ACTGCGTCTTACACTTGGCA |
| Sirt7 H | TTGGTCGTCTACACAGGCG | | | CAGACGGGTGATGCTCATGT |

M:Mouse H:Human

**Table S2. The primary and secondary antibodies used for western blot**

| Antibody Name | Antibody ID | Source |
| --- | --- | --- |
| EpCAM | ab71916 | Abcam |
| GAPDH | #2118 | CST |
| GAPDH | ab9485 | Abcam |
| p-EGFR | #3777 | CST |
| EGFE | ab32562 | Abcam |
| p-SP1 | PA5-1004771 | Thermo Fisher Scientific |
| SP1 | #9389 | CST |
| TERT | NB100-317 | Novus Biologicals |
| γH2AX | ab26350 | Abcam |
| H2AX | ab217838 | Abcam |
| SIRT7 | #5360 | CST |
| SIRT6 | #12486 | CST |
| SIRT5 | #8779 | CST |
| SIRT4 | PA5-83096 | Invitrogen |
| SIRT3 | #5490 | CST |
| SIRT2 | sc-28298 | Santa Cruz Biotechnology |
| SIRT1 | #8469 | CST |
| TSC2 | ab32554 | Abcam |
| TSC1 | ab227594 | Abcam |
| P-mTOR | #2971 | CST |
| mTOR | #2972 | CST |
| P-S6 | #5364 | CST |
| S6 | #2217 | CST |
| P-4EBP1 | #2855 | CST |
| 4EBP1 | #9644 | CST |
